# MicroRNA regulation of endothelial TREX1 reprograms the tumour microenvironment

**DOI:** 10.1038/ncomms13597

**Published:** 2016-11-25

**Authors:** RaeAnna Wilson, Cristina Espinosa-Diez, Nathan Kanner, Namita Chatterjee, Rebecca Ruhl, Christina Hipfinger, Sunil J. Advani, Jie Li, Omar F. Khan, Aleksandra Franovic, Sara M. Weis, Sushil Kumar, Lisa M. Coussens, Daniel G. Anderson, Clark C. Chen, David A. Cheresh, Sudarshan Anand

**Affiliations:** 1 Department of Cell, Developmental and Cancer Biology, Department of Radiation Medicine, Oregon Health and Science University, Portland, Oregon 97229, USA; 2 Department of Radiation Medicine and Applied Sciences, University of California, San Diego, California 92093, USA; 3 Department of Neurosurgery, University of California, San Diego, California 92093, USA; 4 Department of Chemical Engineering, Institute for Medical Engineering and Science, David H Koch Institute for Integrative Cancer Research, Massachusetts Institute of Technology, Cambridge, Massachusetts 02139, USA; 5 Department of Pathology at the UC San Diego Moores Cancer Center and Sanford Consortium for Regenerative Medicine, University of California, San Diego, La Jolla, California 92037, USA

## Abstract

Rather than targeting tumour cells directly, elements of the tumour microenvironment can be modulated to sensitize tumours to the effects of therapy. Here we report a unique mechanism by which ectopic microRNA-103 can manipulate tumour-associated endothelial cells to enhance tumour cell death. Using gain-and-loss of function approaches, we show that miR-103 exacerbates DNA damage and inhibits angiogenesis *in vitro* and *in vivo*. Local, systemic or vascular-targeted delivery of miR-103 in tumour-bearing mice decreased angiogenesis and tumour growth. Mechanistically, miR-103 regulation of its target gene *TREX1* in endothelial cells governs the secretion of pro-inflammatory cytokines into the tumour microenvironment. Our data suggest that this inflammatory milieu may potentiate tumour cell death by supporting immune activation and inducing tumour expression of Fas and TRAIL receptors. Our findings reveal miR-mediated crosstalk between vasculature and tumour cells that can be exploited to improve the efficacy of chemotherapy and radiation.

## Abstract (web summary)

The tumour microenvironment can be modulated to sensitize tumours to the effects of therapy. Here the authors show that radiation induced miR-103 downregulates TREX1 in endothelial cells, decreases angiogenesis and leads to the secretion of proinflammatory mediators that reduce tumour growth.

## Graphical abstract

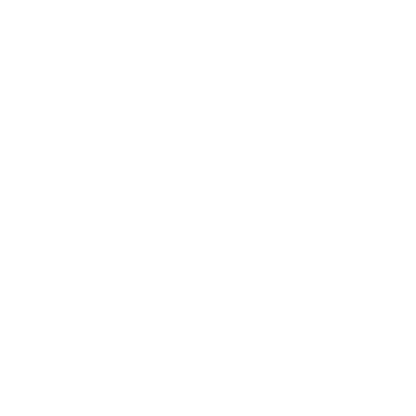

Vascular endothelium is a highly complex, dynamic organ with a growing list of sophisticated functions. As such, the endothelial cells (ECs) encounter a variety of insults and injuries during pathogenesis and have developed intricate stress response pathways that enable cell survival decisions. Intriguingly, emerging studies suggest that DNA damage response pathways (DDR) in ECs play a pro-angiogenic role^1^,^2^. The histone H2AX and an efficient DDR were shown to be important for EC proliferation under hypoxia and hypoxia-related angiogenesis^1^. Similarly, the ataxia telangiectasia-mutated kinase, a master regulator of the DDR drives pathological angiogenesis^2^. Given the critical role of hypoxia, oxidative stress and DNA damage in the tumour microenvironment, understanding the molecular basis of how ECs deal with these stressors will enable the development of a new class of DDR-targeted therapies for regulation of angiogenesis. Indeed, it has been proposed that EC death is one of the critical determinants of radiation damage^3^,^4^, a key therapeutic modality in cancer treatment. Since miRs influence several aspects of endothelial function^5^, we hypothesized that miRs regulate endothelial apoptosis in response to radiation damage. We have identified a microRNA (miR) signature in ECs induced in common by genotoxic stress, oxidative stress and DNA damage. We demonstrate that the most upregulated miR in this signature, miR-103, is a negative regulator of EC DNA repair, cell survival and sprouting angiogenesis *in vitro*. Importantly, we observe that exogenous miR-103 expression inhibits both developmental and pathological angiogenesis *in vivo*. We show that miR-103 targets non-canonical DNA repair enzymes, three prime exonucleases (TREX) 1 and 2 in ECs. MiR-103 regulation of TREX1 induces several pro-inflammatory cytokines and a type I interferon response *in vitro*. Indeed, vascular-targeted delivery of miR-103 induces proinflammatory cytokines, costimulatory molecules and decreases PD-L1+ macrophages and neutrophils in the tumour microenvironment. Taken together, our data identifies miR-103 regulation of TREX1 as a potent modulator of the tumour microenvironment.

## Results

### DDR induces miR-103 and miR-103 exacerbates DNA damage

**Figure 1:**
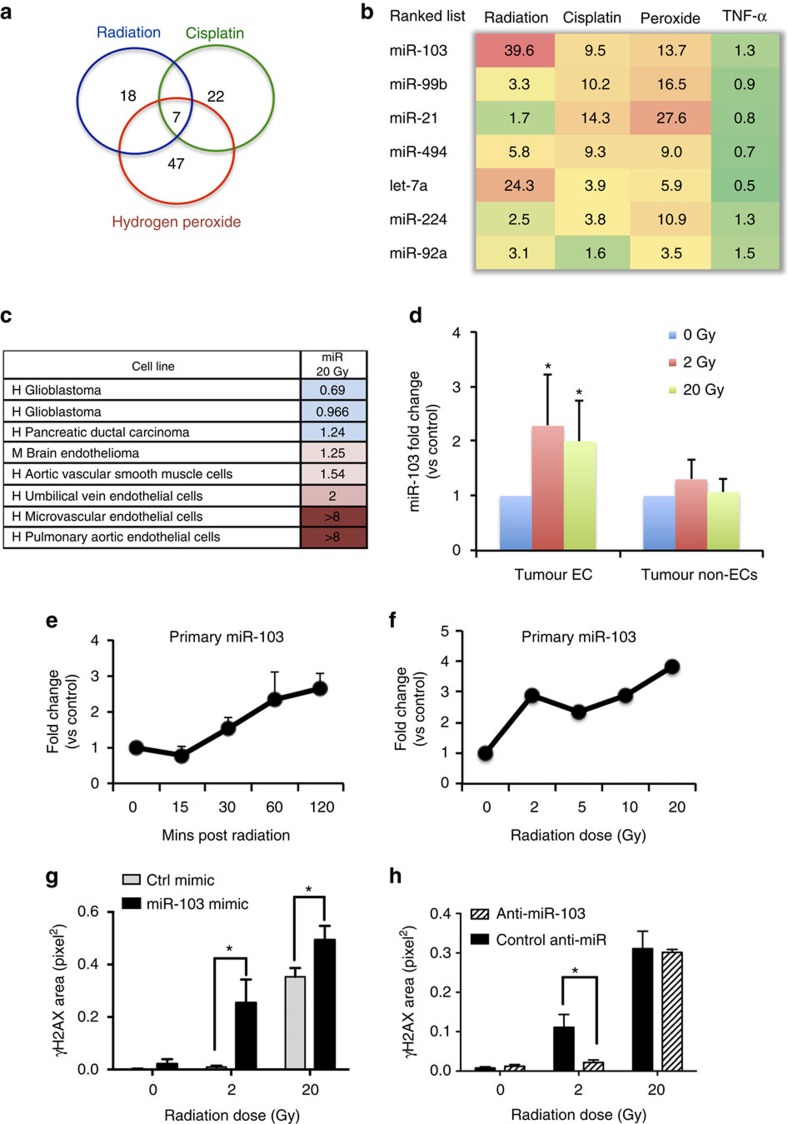
miR-103 is upregulated during DNA damage and exacerbates DNA ds breaks. (**a**) Venn diagrams showing miR profiling results from HUVECs treated with the indicated genotoxic agents and (**b**) expression levels of the top seven upregulated miRs (at least 1.5-fold cutoff). The numbers indicate fold change over control treated HUVECs normalized to a housekeeping small RNA at 6 h post treatment. (**c**) Heatmap depicting expression of miR-103 in different vascular cell types 6 h after radiation. Numbers depict mean fold increases over mock-treated cells. (**d**) qRT-PCR showing the expression of miR-103 in ECs 3 h post radiation in a 4T1 mammary carcinoma model. *N*=3 mice per group and two tumours per mouse. Bars show mean+s.e.m. * indicates *P*<0.05 by two-tailed Student's *t*-test. (**e**) qRT-PCR assay showing induction of miR-103 transcript (pri-miR) in HUVECs over time after a single 20 Gy dose of radiation. Mean+s.e.m. of two biological replicates is shown (**f**) qRT-PCR of pri-miR-103 in HUVECs 1 h after indicated doses of radiation. One of two representative experiments (**g**,**h**) Phosphorylation of histone H2AX in HUVECs measured by immunofluorescence staining 24 h after ectopic expression of miR-103 (**g**) and inhibition of miR-103 (**h**) 3 h after exposure to the indicated doses of radiation. Bars show mean+s.e.m. of pixel area normalized to the number of nuclei for 25–50 nuclei per group. * indicates *P*<0.05 by two-tailed Student's *t*-test. qRT-PCR; quantitative real-time PCR.

We observed that silencing of the microRNA processing enzyme Dicer in human umbilical vein ECs significantly increased DNA damage in these cells as measured by a Comet assay (Supplementary Fig. 1). We reasoned that individual miRs capable of inducing a DDR in vascular cells may represent a powerful therapeutic strategy to sensitize tumours to the effects of therapy. To identify specific miR(s) involved in this function, we screened for miRs that were differentially expressed in ECs 6 h after irradiation or treatment with cisplatin or hydrogen peroxide (Supplementary Fig. 2) and found miR-103 as the top candidate among a set of seven miRs upregulated in response to all three inducers of intrinsic apoptosis (Fig. 1a,b). We first asked if miR-103 upregulation was a typical response to cell stress across a panel of cell types. Surprisingly, we found that miR-103 was preferentially upregulated in primary vascular cells including ECs and vascular smooth muscle cells but not in a range of other cells such as pancreatic and brain tumour cells (Fig. 1c). Indeed, irradiation of an orthotopic 4T1 mammary carcinoma resulted in rapid upregulation of miR-103 in the tumour ECs but not in the non-EC fraction (Fig. 1d). Moreover, we found that primary miR-103 was transcribed as early as 30 min after radiation (Fig. 1e) and in response to a range of doses from 2 to 20 Gy (Fig. 1f and Supplementary Fig. 3). We then asked whether miR-103 had a functional role in response to DNA damage induced by radiation. Indeed ectopic expression of miR-103 mimic exacerbated DNA double strand (ds) breaks induced by radiation as measured by phosphorylation of histone H2AX, while inhibition of miR-103 decreased the ds breaks induced by radiation (Fig. 1g,h and Supplementary Fig. 4a). Consistently, miR-103 induced exacerbation of DNA damage in the presence of radiation was also apparent in a neutral comet assay (Supplementary Fig. 4b).

### miR-103 induces EC death and inhibits sprouting angiogenesis

**Figure 2:**
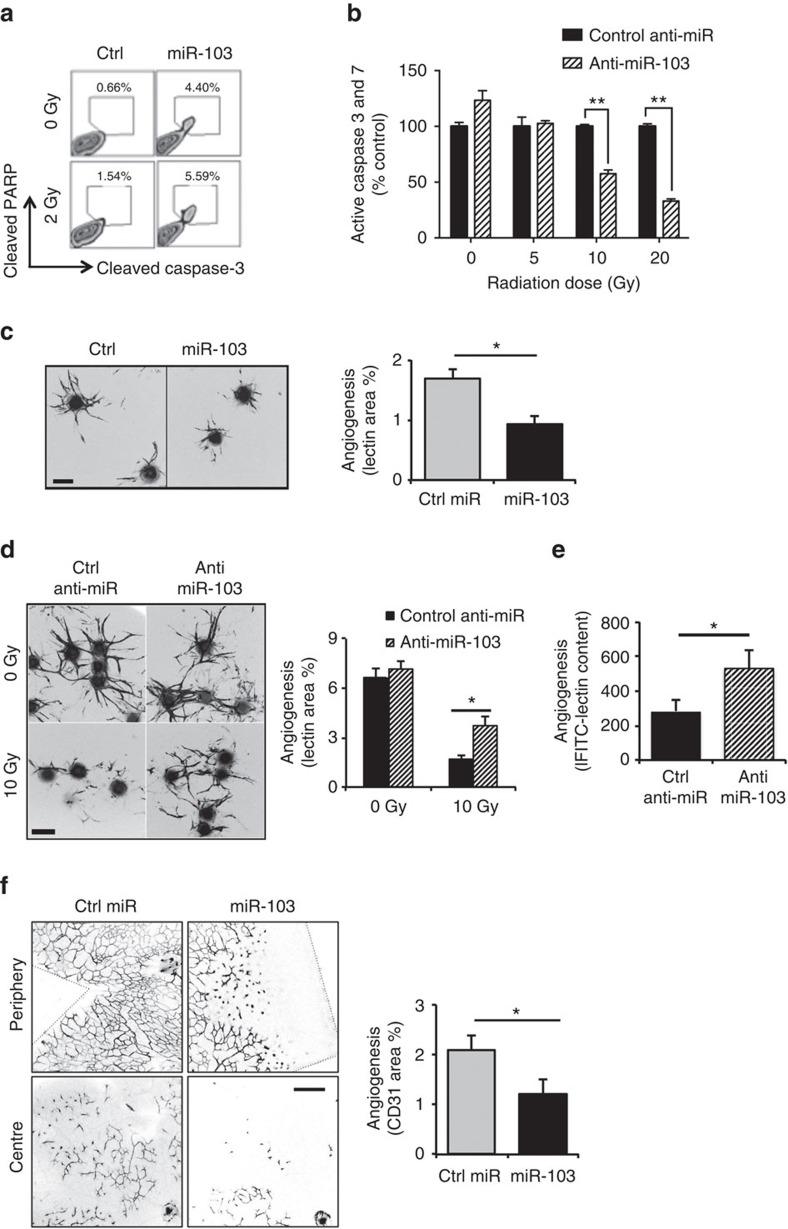
miR-103 induces EC death and decreases sprouting angiogenesis *in vitro* and *in vivo*. (**a**,**b**) Apoptosis, as measured by cleaved PARP and caspase-3, 48 h after ectopic expression of miR-103 (**a**) or (**b**) a caspase Glo- luminescence assay for active caspases 3 and 7 after ectopic expression of miR-103 in HUVECs 48 h after exposure to the indicated doses of radiation. One out of three independent experiments. Bars show mean+s.e.m. ** indicates *P*<0.01 by two-tailed Student's *t*-test. (**c**) 3D angiogenic sprouting assay with expression of miR-103. Right panel bars show mean+s.e.m. of lectin stained sprout areas from at least 10 beads per group. *P<0.05 by two-tailed Student's *t*-test. Scale bar, 100 μm. One of four independent experiments. (**d**) 3D angiogenic sprouting assay with expression of Anti-miR-103. Right panel bars show mean+s.e.m. of lectin stained sprout areas from at least 10 beads per group. *P<0.05 by two-tailed Student's *t*-test. Scale bar, 100 μm. One of three independent experiments. (**e**) Quantification of angiogenesis in mouse Matrigel plugs after i.v injection of control or miR-103 inhibitor followed by 10 Gy local radiation (*N*=4–6 plugs per group, one out of the three independent experiments). Bars show mean+s.e.m. * indicates *P*<0.05 by two-tailed Student's *t*-test. (**f**) Whole mount images of mouse neonatal P12 retina stained with anti-CD31 5d post intraocular injection with miR-103. Right panel bars show mean+s.e.m. of CD31 area from *n*=5 retinas. *P<0.05 by two-tailed Student's *t*-test. Scale bar, 200 μm.

We then asked if there were functional consequences due to miR-103-mediated DNA damage in ECs. Surprisingly, transfection of miR-103 in ECs significantly increased markers of apoptosis, cleaved caspase-3 and poly (ADP-ribose) polymerase (PARP) even in the absence of radiation and in combination with a single dose of radiation (Fig. 2a and Supplementary Fig. 5) suggesting that miR-103 functions as a pro-apoptotic miR (apoptomiR) in ECs. Interestingly, the apoptotic effects of miR-103 were lost upon EC culture in minimal growth conditions in basal medium (Supplementary Fig. 6) suggesting that this miR may be apoptotic only to proliferating ECs. Conversely, inhibition of miR-103 decreased the activation of caspases 3 and 7 induced by radiation (Fig. 2b). To further dissect the physiological consequences of miR-103, we examined its activity in a three-dimensional sprouting angiogenesis assay^6^. Consistent with its pro-apoptotic function, miR-103 expression resulted in a 50% decrease in endothelial sprouting (Fig. 2c). Inhibition of endogenous miR-103 induced by radiation rescued sprouting angiogenesis *in vitro* (Fig. 2d) and in an FGF Matrigel plug angiogenesis model *in vivo* (Fig. 2e and Supplementary Fig. 7). Having established the anti-angiogenic role of miR-103 *in vitro*, we asked if it had an effect *in vivo* using a developmental angiogenesis model in neonatal mouse retinas^7^. Intraocular injection of miR-103 into postnatal day 5 pups decreased retinal neovascularization in the deep plexus by 50% over a control miR (Fig. 2f). These observations led us to confirm that miR-103 is not only expressed in response to genotoxic stress but also functions as an apoptomiR that can suppress angiogenesis *in vitro* and *in vivo*.

### miR-103 diminishes angiogenesis and tumour burden

**Figure 3:**
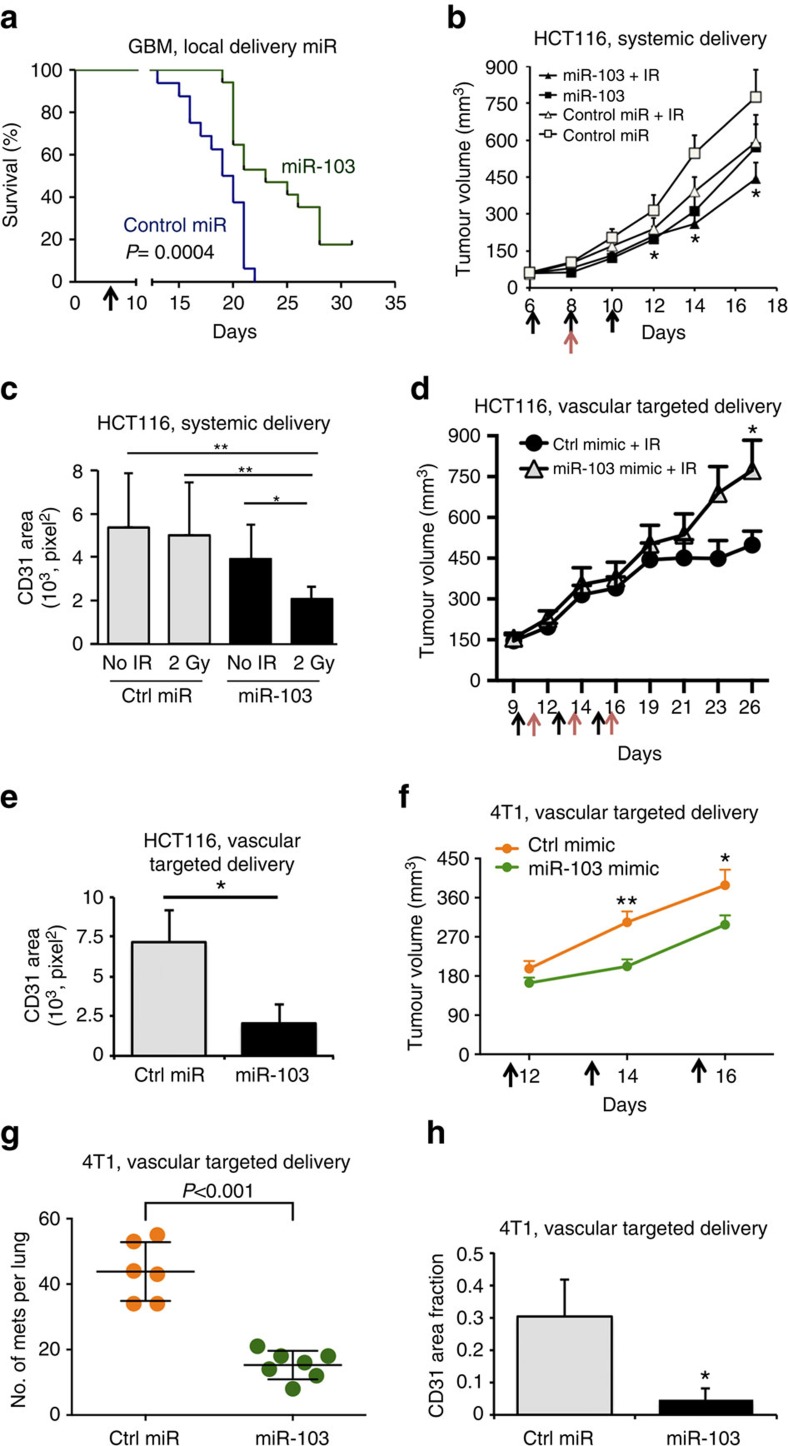
miR-103 decreases angiogenesis and tumour burden. (**a**) GBM-NS-001 cells (2 × 10^4^) were orthotopically implanted into brains of nude mice. miRs were injected locally 7 days post tumour implantation (*N*=14 mice per group). *P* value from a log-rank (Mantel–Cox) test. (**b**) HCT116 colon carcinoma cells were implanted subcutaneously in nude mice. Mice were treated with 50 μg of either control miR or miR-103 mimic on days 6, 8 and 10 (black arrows). Mice received a single dose of 2 Gy radiation on day 8 (red arrows). Mean tumour volume measurements of *N*=8 mice per group are shown. Error bars represent s.e.m. *P<0.05 by Mann–Whitney *U*-test. (**c**) Angiogenesis was measured by staining tumour sections with anti-CD31. Quantification of CD31 area from at least three mice per group are shown. Bars show mean+s.e.m. *P<0.05, **P<0.01. (**d**) HCT cells were implanted as in **b**. (*N*=8 mice per group) Mice were treated with either a control mimic or mir-103 mimic in 7C1 nanoparticles (0.7 mg kg^−1^ of miR, i.v.) on days 9,12 and 15 (black arrows) and were irradiated locally on days 10,12 and 16 (red arrows). Mean tumour volume measurements of *N*=8 mice per group are shown. Error bars represent s.e.m. *P<0.05 by Mann–Whitney *U*-test. (**e**) Angiogenesis was measured as in **c**. Bars show mean+s.e.m. *P<0.05 by two-tailed Student's *t*-test. (**f**,**g**) 4T1 mouse mammary carcinoma cells (1 × 10^4^) were implanted in the mammary fat pads of 6–8 week old female Balb/C mice (*N*=6 controls, 7 treated). Mice were randomized to receive either Control miR or miR-103 mimic formulated as miR-7C1 nanoparticles (0.7 mg kg^−1^, i.v.) on days 12, 14, 16. Primary tumour volume measurements and gross metastatic foci per lung on day 20 are shown. Error bars depict s.e.m. *P<0.05 and **P<0.01 by Mann–Whitney *U*-test. (**h**) Angiogenesis was measured by staining the 4T1 tumour sections with anti-CD31. Bars show CD31 area normalized the tumour area as mean+s.e.m. from *n*=4 tumours per group. *P<0.05 by two-tailed Student's *t*-test.

We sought to address if miR-103 is sufficient to decrease pathological angiogenesis and tumour burden *in vivo*. Since anti-angiogenic agents have been successful in the treatment of glioblastomas and colorectal carcinomas^8^,^9^,^10^, we chose to investigate the effects of miR-103 in mouse xenograft models of these two tumour types. Strikingly, local delivery of miR-103 a week after tumour implantation increased survival in an aggressive orthotopic glioblastoma model (Fig. 3a). Moreover, treatment of subcutaneous HCT-116 colon carcinoma xenografts with three doses of a miR-103 mimic and a single dose of radiation significantly decreased tumour burden (Fig. 3b) when compared with mice that received a control mimic. Consistent with our *in vitro* observations on angiogenic sprouting, there was a 60% decrease in vascular area (CD31 staining) in mice that received both miR-103 and radiation (Fig. 3c) a week after the last miR-103 injection. To address whether this effect was specifically due to the action of miR-103 on the tumour endothelium, we utilized a recently characterized^11^,^12^ EC targeted nanoparticle 7C1 to deliver miR-103. We observed that intravenous injection of 7C1-miR-103 resulted in an approximately threefold enrichment of miR-103 in tumour ECs but not in the non-EC fraction (Supplementary Fig. 8). Delivery of a 10-fold lower dose of miR-103 mimic (0.7 mg kg^−1^) alternating with a 2 Gy dose fraction of radiation was sufficient to decrease tumour burden (Fig. 3d) concomitant with a significant decrease in CD31 area (Fig. 3e). It has been observed that anti-angiogenic agents can potentially cause an increase in metastasis in breast cancer^13^,^14^. Therefore, we asked if our miR-103 treatment could impact growth and/or metastasis of a highly aggressive triple negative mammary carcinoma 4T1 in syngeneic Balb/C mice. We found that while miR-103 had a modest but measurable effect on tumour burden (Fig. 3f), there was a significant decrease in the number of lung metastases (Fig. 3g). Indeed, consistent with our other tumour models, we also observed a decrease in CD31 area (Fig. 3h) indicating the impact of the miR treatment on angiogenesis. Our observations with both gain-and-loss of function of miR-103 *in vitro* and in developmental and pathological angiogenesis models *in vivo* argue that miR-103 decreases angiogenesis.

### miR-103 targets DNA repair pathway enzymes

**Figure 4:**
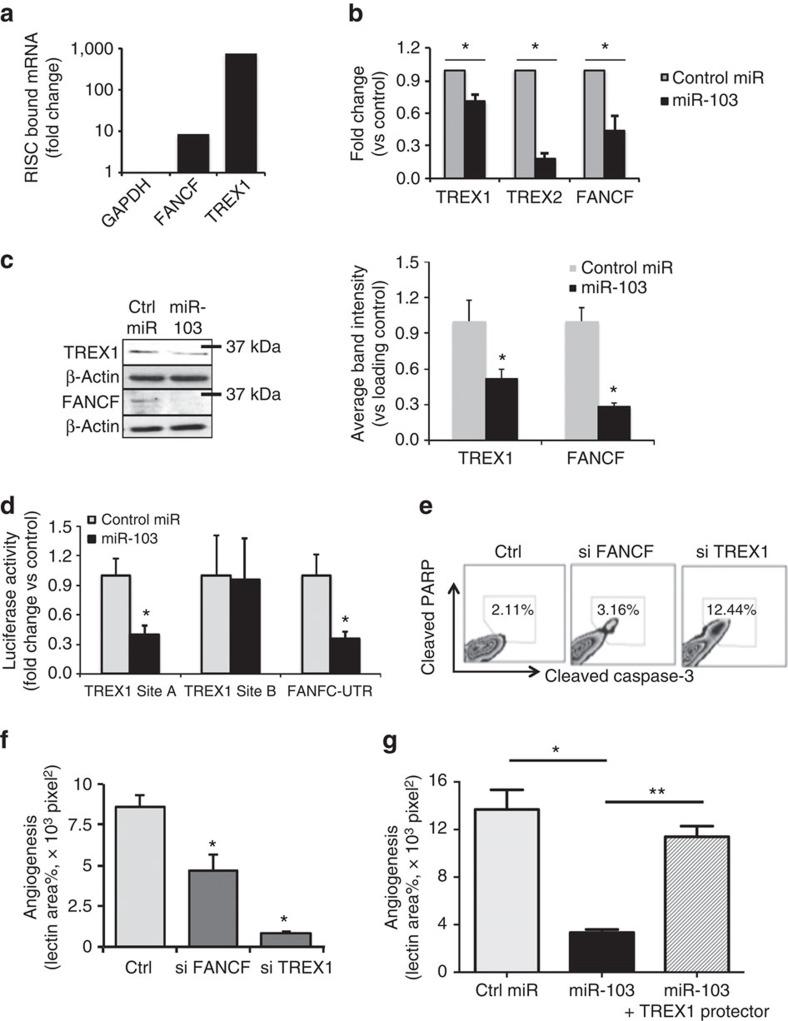
miR-103 regulates DNA repair enzymes TREX1 and FANCF to decrease angiogenesis. (**a**) Mir-TRAP assay depicting fold enrichment of miR-103 targets 24 h after miR-103 transfection and pull down of RISC relative to control miR. One out of three independent experiments (**b**) qRT-PCR (24 h) and (**c**) western blot (48 h) from HUVECs transfected with miR-103 showing levels of TREX1 and FANCF. Right panel shows quantitation of blots from two independent experiments. *P<0.05 by two-tailed Student's *t*-test. (**d**) Luminescence from 3′-UTR-luciferase constructs with indicated miR-103 binding regions 24 h after transfection with miR-103. *P<0.05 by two-tailed Student's *t*-test. One out of two independent experiments. (**e**) Apoptosis as measured by cleaved PARP and caspase-3 48 h after knockdown of FANCF, TREX1. One of four independent experiments. (**f**) 3D angiogenic sprouting assay after knockdown of FANCF, TREX1. *P<0.05 by two-tailed Student's *t*-test. One out of three independent experiments. *P<0.05 by two-tailed Student's *t*-test. (**g**) 3D sprouting assay using HUVECs transfected with indicated oligos. All bars show mean+s.e.m. *P<0.05, **P<0.01 by ANOVA. 3D, three-dimensional; ANOVA, analysis of variance; qRT-PCR, quantitative real-time PCR.

miRs are thought to exert significant effects on gene expression programs by binding to mRNAs, generally at their 3′-untranslated region (3′-UTR) and recruiting them to an RNA-induced silencing complex (RISC) for degradation^15^. To investigate the target(s) of miR-103 relevant to its role as an apoptomiR in ECs, we compared mRNA expression levels for 92 genes using a Taqman DNA damage-related gene signature panel for ECs that were treated with radiation alone or transfected with miR-103 mimic (Supplementary Fig. 9). Surprisingly, only three mRNAs were downregulated in both groups—the 3′-exonucleases *TREX1* and *TREX2* and the fanconi anaemia complementation group F gene, *FANCF*. Mutations in *TREX1* have been associated with several human diseases including systemic lupus erythematosus and retinal vasculopathy with cerebral leukodystrophy (RVCL)^16^,^17^,^18^. Coincidentally, RVCL has been described as a disease with loss of capillaries in the retina and the brain pathology has been noted to be similar to radiation damage^19^. We utilized a RISC-Trap assay^20^ and observed that miR-103 transfection resulted in robust enrichment of *TREX1* and a modest level of *FANCF* transcripts associated with the RISC (Fig. 4a). Human *TREX1* mRNA has a short 3′-UTR that does not contain any putative miR-103-binding sites. RNA hybrid modelling identified two high-affinity binding sites A and B in the coding region of *TREX1* (Supplementary Fig. 10). *FANCF* on the other hand has one miR-103 binding site in the 3′UTR (Supplementary Fig. 10). Consistent with the enrichment in the RISC-Trap assay, ectopic expression of miR-103 decreased both mRNA and protein levels of TREX1, TREX2 and FANCF (Fig. 4b,c and Supplementary Fig. 11). Moreover, one of the *TREX1* sites, site A and the *FANCF* site decreased the expression of an upstream luciferase by more than 60% (Fig. 4d). Mutations that disrupted miR-103 binding on *TREX1* site A restored the luciferase levels (Supplementary Fig. 12) indicating that miR-103 directly binds to *TREX1* in this region. Indeed, knockdown of TREX1 recapitulated the miR-103-induced apoptosis (Fig. 4e) and sprouting angiogenesis (Fig. 4f), whereas the silencing of FANCF had more modest effects. Importantly, expression of a target protector, an oligonucleotide sequence that is complementary to the *TREX1* miR-103 binding site but specific to *TREX1* was able to rescue miR-103 mediated decrease in sprouting angiogenesis (Fig. 4g) indicating the decrease of TREX1 is the major mechanism by which miR-103 mediates EC death and suppresses angiogenesis. Finally, we examined TREX1 and FANCF levels in the tumour ECs from a xenograft GBM model and found significant downregulation of both at the RNA level (Supplementary Fig. 13). Similarly 4T1 tumours treated with our EC targeted miR-103 had significant downregulation of TREX1 staining in the tumour endothelium (Supplementary Fig. 13).

### miR-103 inhibition of TREX1 induces interferons

**Figure 5:**
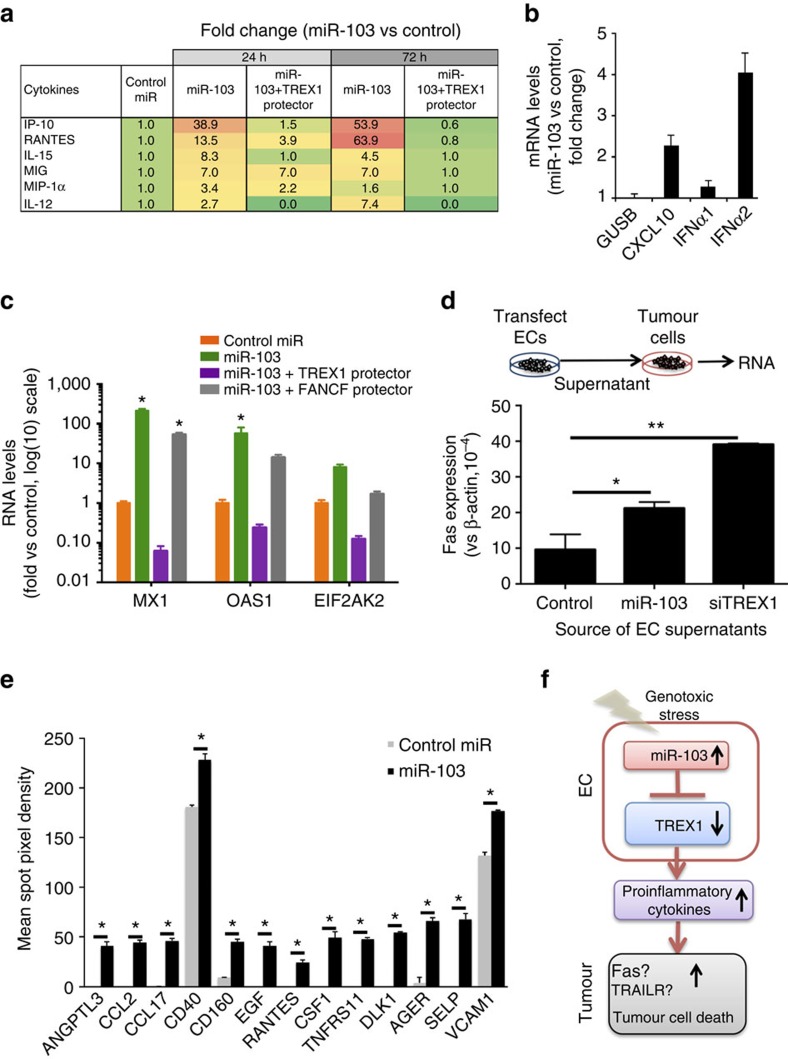
miR-103 treatment induces an inflammatory secretome *in vitro* and *in vivo*. (**a**) Cytokines upregulated at least twofold in HUVECs transfected with miR-103 or miR-103 in the presence of a TREX1 target protector. Mean fold change of two independent biological replicates vs control miR in a multiplex cytokine assay are depicted. (**b**) qRT-PCR of *IP-10 (CXCL10)* and *IFNα1* and *IFNα2* from HUVECs transfected with a control mimic or miR-103 mimic. Fold change at 24 h post transfection compared with control mimic is depicted. Bars show mean+s.e.m. One out of three independent experiments. (**c**) mRNA levels of canonical interferon-stimulated genes *MX1, OAS1* and *EIF2AK2* from HUVECs transfected with the indicated micro-RNAs and target protector oligos. Bars show mean+s.e.m. *P<0.05 by two-tailed student's *t*-test compared with control miR. (**d**) Fas expression on MDA-MB-231 human breast cancer cells 24 h after exposure to conditioned media from HUVECs transfected with the indicated RNAs. Bars show mean fold change+s.e.m. *P<0.05, **P<0.01 by ANOVA. One of three independent experiments. (**e**) Quantitation of protein expression from 4T1 tumour lysates from either control treated mice or miR-103 treated mice (experiment in Fig. 3f) using a western blot membrane array. Mean spot density of two independent tumours is shown. Significantly different proteins (*P<0.01 by two-tailed Student's *t*-test) are depicted. Bars show mean+s.e.m. (**f**) Schematic representation depicting putative mechanism of action of miR-103. ANOVA, analysis of variance; qRT-PCR, quantitative real-time PCR.

Since loss of TREX1 has been linked to accumulation of ssDNA, defective DNA repair and elaboration of cytokines^21^,^22^, we asked if miR-103 regulated the expression of cytokines using a multiplex chemokine/cytokine assay. Indeed, miR-103 expression significantly upregulated proinflammatory chemokines IP-10, RANTES, MIG and cytokines IL-15, IL-12 and IFN-γ (Fig. 5a). Importantly, the miR-103 mediated induction of these factors was diminished in the presence of a TREX1 target protector (Fig. 5a and Supplementary Fig. 14) indicating that these chemokines and cytokines are specifically regulated by miR-103 mediated decrease in TREX1. The most upregulated chemokine in our miR-103 supernatants, IP-10 has recently been shown to be induced in lymphoblasts from patients with RVCL and in TREX1 V235fs mutation knock-in mice^23^. Consistent with our *in vitro* data, we observed a significant increase in *IP-10* mRNA levels in tumour ECs from our 7C1-miR-103 treated mice (Supplementary Fig. 15a). IP-10 is a well-known negative regulator of angiogenesis *in vitro* and *in vivo*^24^,^25^,^26^. Our cytokine profile also indicates coordinated regulation of related cytokines. For example, IL-12 is known to elevate the production of IFN-γ which, in turn, induces the production of IP-10 and MIG^27^. Some of these cytokines, that is, IL-12 and IFNs are also regulators of cell death pathways. Indeed, we found that miR-103 induced both *IP-10* and *IFNα2* (Fig. 5b). In fact, we found there was a robust upregulation of canonical interferon stimulated genes *MX1* and *OAS1* (Fig. 5c). We found that these increases were dependent on TREX1 and not FANCF (Fig. 5c). Therefore, we hypothesized that the cytokine milieu from the ECs expressing miR-103 may also promote tumour cell death in a paracrine fashion. To test this hypothesis, we used an apoptosis protein array and found that miR-103 treatment in mice led to a significant upregulation of several pro-apoptotic pathways in the HCT-116 tumours, particularly a ∼30-fold increase in the levels of Fas and TRAIL-Rs (Supplementary Fig. 15b). Indeed, conditioned media from miR-103 transfected ECs upregulated HCT-116 expression of *Fas* and *TRAIL* receptors in a TREX1-dependent manner (Supplementary Fig. 15c). Similarly, conditioned media from either miR-103 transfected ECs or TREX1-silenced ECs upregulated *Fas* transcription in MDA-MB-231 breast cancer cells (Fig. 5d). Finally, inhibition of Fas with an antagonistic antibody decreased the miR-103-mediated activation of Caspase-8 (Supplementary Fig. 15d). Our data suggests that radiation induced miR-103 downregulates TREX1 in ECs, decreases angiogenesis and leads to the secretion of proinflammatory mediators that may upregulate Fas and TRAIL pathways in tumour cells. We tested this hypothesis by interrogating the 4T1 tumour lysates from mice treated with control miR or miR-103 (described in Fig. 3f,g) using a proteome profiler western blot membrane array. In addition to key chemokines, we also saw a significant increase in costimulatory receptors CD40, CD160, adhesion molecules and receptors for advanced glycation end products (Fig. 5e). Interestingly, we also saw a decrease of the immune checkpoint ligand PD-L1 expression on macrophages and neutrophils in the tumours (Supplementary Fig. 16) as well as a decrease in the numbers of these immunosuppressive cells in the tumours^28^ suggesting that miR-103 treatment may modulate the immune microenvironment.

## Discussion

Our findings demonstrate that miR-103 is a potent apoptomiR that can decrease angiogenesis via regulation of TREX1/FANCF. Previous studies have shown that miR-103 has been shown to be involved in diverse physiological processes including metastasis control^29^, insulin sensitivity^30^ and resistance to EGFR inhibition in lung cancer^31^. Interestingly, Taniguchi and colleagues identified miR-103 as one of the top miRs in a functional screen for DNA damage disrupting agents that affect chemotherapy sensitivity^32^. They identified Rad51 and Rad51D as key targets of miR-103/miR-107 that enabled the effects of the miRs in enhancing chemosensitivity of tumour cells. Chen *et al*.^33^ have shown miR-103/107 are late response genes in hypoxia and function together with Let-7a/7e to regulate AGO1 and VEGF pathways that leads to an increase in angiogenesis. However, since their treatment approaches used a combination of three miR inhibitors (Anti-let7a, let7e and miR-103), the specific role of miR-103 in ECs remains unclear. Consistent with our miR-103 findings, miR-107 which shares the same seed sequence as miR-103, has been reported to decrease tumour angiogenesis^34^. Our gain and loss of function experiments *in vitro* and *in vivo* argue that miR-103 is a negative regulator of angiogenesis.

We have identified a TREX1 as a novel target for miR-103 in endothelial DDRs. While TREX1 has not been considered a canonical DNA repair enzyme, recent studies highlight a broader role for this exonuclease. For example, TREX1 has been shown to interact with PARP and facilitates its nuclear localization and activity^35^. In addition to ssDNA, TREX1 has now been implicated in the degradation of dsDNA and ssRNA^36^,^37^. In fact, TREX1 has even been shown to have nuclease independent activities in the regulation of oligosaccharyltransferase that contributes to its role in immune regulation^23^. Moreover, studies have shown that deregulation of TREX1 can mediate inflammation^38^,^39^. Our observations demonstrate another unique but critical role for TREX1 downstream of miR-103 in promoting EC death and dysfunction in response to radiation. The elaboration of proinflammatory mediators by miR-103 is also dependent on TREX1 and suggests that both cell intrinsic and extrinsic mechanisms play a role in miR-103 function.

It is increasingly clear that the tumour microenvironment, particularly the vasculature plays a critical role in tumour progression and responses to therapeutics^40^,^41^. Our data highlights a novel molecular mechanism that not only regulates EC death in DDRs but also provokes an inflammatory milieu that could both diminish pro tumorigenic cells such as PD-L1+ macrophages and sensitize the surrounding tumour cells to death receptor pathways. We believe these results elucidate a complex crosstalk between endothelial DNA damage and the tumour microenvironment that can be exploited to augment treatment regimens in cancer.

## Methods

### Cell culture and reagents

HUVECs (Lonza) were cultured in EBM-2 media (Lonza) supplemented with bullet kit and 10% fetal calf serum (Hyclone). HCT-116 cells (ATCC), 4T1 cells (ATCC) were culture in McCoy's media, DMEM or RPMI-1640 supplemented with 10% fetal calf serum and antibiotics. Cells were tested and found negative for mycoplasma contamination before use in the assays described.

### Western blotting

Cell and tissue lysates were prepared in RIPA buffer (Pierce 89900) and quantified using a BCA assay (Pierce, #23227) kit. Equivalent amounts of protein were loaded on a 4–12% gradient SDS-polyacrylamide gel (NuPAGE, Life-Technologies) or a 8% SDS–polyacrylamide gel and transferred overnight onto polyvinylidene difluoride (PVDF) membranes. Membranes were blocked in 5% milk or 3% BSA and incubated with antibodies as indicated- Trex1 (Cell Signaling 12215, s 1:1,000 o/n), Fancf (Abnova, h00002188-bo1p 1:1,000 o/n), anti-β-actin antibody (Sigma, A5316, 1:10,000 1 h RT). Membranes were washed in TBST and incubated with secondary antibodies from Licor Biosciences were used goat anti mouse 925-68020 (1:15,000) and goat anti rabbit 925-32211 (1:15,000). Blots were scanned on the Licor Odyssey scanner according to manufacturer's instructions. For specific protein array experiments with membrane arrays -mouse XL cytokine Array (R&D Biosystems-ARY028) and human apoptosis array (R&D Biosystems ARY009), protocols were according to manufacturer's instructions.

### Vectors/plasmids

FANCF Luciferase-3′-UTR plasmid was purchased from SwitchGear Genomics. TREX1 luciferase constructs were cloned by inserting a ∼70 bp fragment of the miR-103-binding region into a pmiR-REPORT vector (Ambion). Luciferase assay reagents were purchased from Switch Gear Genomics.

### miRs/anti-miRs/siRNAs

miR-103 mimics, inhibitors and respective controls were purchased from Life Technologies and Exiqon. For *in vivo* studies, high-performance liquid chromatography-purified miRs were purchased from Life Technologies in bulk quantities. SiRNAs against TREX1 and FANCF were purchased from Life Technologies.

Gapmer TREX1 and TREX1, FANCF target protectors were purchased from Exiqon.

### Transfections

Cells were transfected at 50–60% confluence using standard forward transfection protocols using RNAimax reagent (Life Technologies) for miRs/siRNAs and Lipofectamine 2000 for plasmid or plasmid RNA dual transfections. Typically 50 nM RNA and 1–2 μg plasmid DNA were used for transfections. Target protectors were transfected at a concentration of 50 nM or equivalent to the miR amounts.

### Radiation of cells/mice

Cells or mice were irradiated on a Shepherd ^137^cesium irradiator at a rate of ∼166 cGy min^−1^. In tumour-targeted radiation experiments, mice were restrained in a lead shield (Brain Tree Scientific) to minimize exposure to the non-tumour areas.

### RNA extraction and real time PCR assays

RNA was extracted using miRVana microRNA isolation kit (Ambion) and PCR with reverse transcription (RT–PCR) was performed using multiplexed TaqMan primers (Applied Biosystems). The miR profiles were generated with a 384-well microfluidic card based TaqMan human microRNA panel (Applied Biosystems) amplified on a 7900 HT Fast Real Time PCR system (Applied Biosystems). HUVECs were treated with either a 20 Gy dose of radiation or 10 μM cisplatin or 200 μM hydrogen peroxide or 10ng ml^−1^ of TNF-α for 6 h. At the end of the treatment, RNA was extracted using miRvana microRNA isolation kit (Ambion). 1,000 ng of RNA was reverse transcribed without pre-amplification using the Taqman Megaplex primer pools A and B and real-time PCR was performed on the microfluidic cards for the human microRNA panels A & B per manufacturer's recommendation (Applied Biosystems). Data was normalized to internal control small RNA RNU48 or U6 small RNAs. mRNAs were normalized to either β-actin or GAPDH. Individual RT–PCRs were performed using predesigned TaqMan Assays for mature miRs, primary miRs or mRNAs (Applied Biosystems) on a SmartCycler (Cepheid) or Vii-7 real-time PCR platform (Applied Biosystems) according to manufacturer's instructions.

### Apoptosis assays

HUVECs were transfected with miRs/Anti-miRs using Lipofectamine RNAi Max reagent (Life Technologies). Apoptosis was measured by either a caspase 3/7-luciferase assay kit (Promega) or flow cytometry with anti-cleaved caspase-3 (Cell Signaling Technologies 9579S: 1:200) and anti-cleaved PARP antibodies (Cell Signaling Technologies 5625: 1:200) or western blot for cleaved caspase-3 (Abcam Ab136812 1:300) according to manufacturer's instructions.

### 3D angiogenic sprouting assay

Early passage HUVECs were coated on cytodex-3 (GE Healthcare) beads at a density of 10 million cells per 40 μl beads and incubated in suspension for 3–4 h with gentle mixing every hour. They were plated on TC treated 6 well dishes overnight and resuspended in a 2 mg ml^−1^ fibrin gel with 200,000 human smooth muscle cells. The gel was allowed to polymerize and complete EGM-2 media was added. Sprouts were visualized from days 3 to 4 via confocal imaging after overnight incubation with 1:200 fluorescein isothiocyanate (FITC)-labelled *Ulex europaeus* lectin (Vector labs).

### Comet assay

Ionizing radiation induced DNA double strand breaks were measured using neutral comet tail assay. Cells were irradiated, harvested, and then assayed using the Trevigen CometAssay kit. Comet tail length in pixels was measured using CometScore freeware (TriTek Corp). A total of 50–100 cells were analysed in each sample group.

### γH2AX staining

A total of 100,000 HUVECs were cultured on glass coverslips in 24-well plates and transfected with miRs/siRNAs using RNAimax (Life Technologies). Plates were irradiated 24 h post transfection and cells were fixed at different time points with 4% paraformaldehyde for 10 min at room temperature, permeabilized with 90% methanol for 10 min at 4 °C. Coverslips were blocked with 1.5% normal goat serum and incubated with primary antibody (H2AX Abcam 11174 1: 1,000) in normal goat serum for 1 h, washed and then incubated with secondary antibody for 30 min, washed and then mounted on glass slides for confocal imaging.

### miR *in situ* hybridization

*In situ* hybridization was performed on frozen tumour sections as described by Pena *et al*.^42^ using a DIG labelled miR-103 Locked Nucleic Acid (LNA) probe (Exiqon). DIG was detected by an anti-DIG HRP antibody (Roche) and amplified using a TSA-Plus Cy3 system (Perkin Elmer).

### miR-TRAP/RISC TRAP assay

HUVECs or 293 T cells were co-transfected with a plasmid coding for a flag-tagged dominant negative GW418 mutant (Clontech kit #632016) along with a control mimic or miR-103 mimic according to kit instructions. Twenty-four hours later the RNA protein complexes were crosslinked and the RISC complex was immunoprecipitated using an anti-FLAG antibody and RNA was isolated for quantitative real-time PCR of target genes. The fold enrichment was calculated using pre and post IP controls as well as normalization to the control mimic pull-downs.

### *In vivo* assays

All animal work was approved by the UCSD Institutional Animal Use and Care Committee or OHSU Institutional Animal Use and Care Committee. Immune-compromized 8–10-week-old female nu/nu mice purchased from the UCSD Animal Care Program breeding colony or Jackson Labs were injected subcutaneously with 2 million mycoplasma-negative HCT116 tumour cells in Matrigel (BD). Tumour growth was measured with calipers, with volume computed as ½ × Length × Width^2^. Mice were randomized into groups once the average tumour volume reached 80–150 mm^3^, approximately 6 days after injection. For some experiments, mice were treated with 7C1-nanoparticles containing miR-103 or control miR (0.7 mg kg^−1^, i.v.). GBM-NS-001 cells (2 × 10^4^ cells in 4 ul HBSS) were stereotactically injected into the brains of 5–6-weeks-old nude mice. The coordinates were: 1.8 mm to the right of bregma and 3 mm deep from the dura. miRNA was delivered 7 days post injection. 4T1 cells (1 × 10^4^) were implanted into the mammary fat pad #4 of 6–8 week old female Balb/C mice in 100 μl Matrigel. Mice were randomized into groups once the average tumour volume reached 150 mm^3^, ∼10 days after implantation. Mice were treated with 7C1-nanoparticles containing miR-103 or control miR (0.7 mg kg^−1^, i.v.). Mice were killed ∼day 18–20 for analysis of metastatic burden in lungs. For analysis of immune cells from the tumour microenvironment, mice were perfused with 10–15 ml PBS/heparin before harvest of tumours. Mice with a >20% decrease in body weight were killed and tumours confirmed by histology. For miR measurement studies (Fig. 1d), 4T1 tumours were implanted in the mammary fat pad of Balb/C mice and allowed to grow to a size of ∼300 mm^3^. Tumour-bearing mice were irradiated with the indicated doses (0, 2 or 20 Gy). Mice were killed 3 h post radiation, tumours were harvested for EC isolation followed by quantitative real-time PCR.

### Matrigel plug angiogenesis assay

Growth factor reduced Matrigel (BD) with 400 ng ml^−1^ recombinant human bFGF (Millipore) was injected subcutaneously in Balb/C mice. Mice were injected i.v with 10 μg control or anti-miRs 3 days after plugs were implanted. Four days later mice were irradiated with the indicated doses on a Shepherd ^137^cesium irradiator at a rate of ∼166 cGy min^−1^. 3 days after irradiation mice were injected with 10 μg FITC-conjugated *Griffonia simplificola* lectin, the plugs were harvested, lysed in RIPA-buffer and the FITC content was measured on a spectrophotometer (Glomax, Promega) with a standard FITC filter set.

### Retinal angiogenesis assay

Retinal neovascularization studies were performed as described elsewhere^43^. Briefly, 5 μg of either control mimic or miR-103 mimic was injected into the vitreous cavity of 6-day-old neonatal Balb/C mice under topical analgesia. 6 days later mice were killed, the retinas were dissected, fixed and stained with anti-CD31 antibodies (BD Pharmingen 553372, 1:500 overnight at 4 °C).

### Endothelial cell sorting and RT–PCR

Five to six-week-old Nu/Nu mice were injected with GBM-NS-001 cells (2 × 10^6^) subcutaneously. 20 days post injection, mice were randomized to two groups—control miRNA (*n*=5) or miR103 (*n*=5). 15 nmoles of miR was delivered by tail vein injection. 48 h later, tumours were harvested followed by enzymatic digestion and ECs were isolated as CD45-CD31+CD34+ cells by flow cytometry cell sorting. RNA was isolated from the ECs and RT–PCR was performed to determine the gene expression. Primers are listed as below

*FANCF* forward 5′-CGCTGGCTTCTCGAAAGTC-3′ reverse 5′-CCAGGCACCCTTTTGCAGAT-3′

*TREX1* forward 5′-CGTCAACGCTTCGATGACAAC-3′ reverse 5′- CTCAGCCTAGCAAGCTCTGT-3′

*GAPDH* forward 5′-ATCATCCCTGCCTCTACTGG-3′ reverse5′-GTCAGGTCCACCACTGACAC-3′

### Endothelial cell isolation

Tumours were harvested from mice and mechanically digested using razor blade. Tissue was incubated in collagenase type I (1 mg ml^−1^) (Worthington, #ls004214) for 1 h at 37 °C. The reaction was stopped with EGM2 media 10% fetal bovine serum (FBS) and the solution was filtered with a 70-μm cell strainer. Cells were resuspended in 1 ml of cold media and incubated at 4 °C for 20 min. After that time cells were incubated with CD31 antibody (Genetex Gtx54379 1:400 in PBS) during 30 min at 4 °C with shaking. Cells were incubated with anti-Rat-IgG magnetic beads (1:50 in EGM2) for 30 min. CD31-positive cells were selected with a magnetic rack and kept in RNA later until isolation.

### Single cell isolation

Mice were perfused with a 15 ml solution of PBS/Heparin. Tumour were harvested and and mechanically digested. Tissue was incubated in collagenase type II (2 mg ml^−1^) during 1 h at 37 °C. The reaction was stopped with DMEM 10% FBS and the solution was filtered with a 70 μm cell strainer. Cells were frozen in DMEM 10% DMSO and 1 mg ml^−1^ BSA until the staining for FACS analysis.

### Single cell staining

Cells were plated on a 96-well plate (2 × 10^6^ cells per well), blocked with Fc-Block (BD Bioscience, cat # 553142) 1:200 and Live/ Death Aqua reagent solution 1:500 for 25 min on ice. Subsequently cells were stained with an antibody cocktail mix containing the following antibodies with the indicated fluorophores—CD11C (563057, BD Biosciences, BV605 1:200), CD11B (563401, BD Biosciences, BV771 1:400), PDL1 (12-5982, eBioscience, PE 1:200), CD45 (25-0451, eBioscience, PE-Cy7 1:500), F4/80 (17-4801-80, eBioscience, APC 1:300) and Ly6G (127623, Biolegend APC-Cy7 1:200) in FACS buffer (PBS 2% FBS and 1 mM EDTA) for another 25 min. After external staining cells were fixed with Cytofix Buffer (BD Bioscience, cat # 554655). After the staining cells, were washed and resuspended in 200 ul of FACS buffer and stored at 4 °C protected from light until analysis in a BD-LSR-Fortessa cell analyser. Data was analysed with FACSDIVA software.

### Immunofluorescence and microscopy

In some experiments, TREX1 was visualized using immunofluorescence staining from OCT sections of tumour tissue. Slides were fixed with 4% PFA and stained overnight for Trex1 (BD bioscience 611986 1:200 o/n). Imaging was performed on a Nikon Spectral C1 confocal microscope (Nikon C1si with EZC1 acquisition software, Nikon Instruments) with Plan Apo 10X/0.45 air, Plan Apo 20X/0.75 air, and Plan Apo 60X/1.40 oil objective lenses (Nikon). Some immunofluorescence imaging was performed on a Yokogawa CSU-W1 spinning disk confocal microscope with × 20 0.45 Plan Fluor objective (Nikon). All images were taken with a channel series. Images were analysed with Image J software for determination of CD31 or lectin area.

### Luminex cytokine panels

Cells were transfected with the indicated oligos, then the supernatants were removed at 24, 48 or 72 h and analysed on a Luminex Human Cytokine Magnetic 25-Plex Panel (Part No. LHC0009M) per manufacturer's instructions.

### Statistics

All statistical analysis was performed using Excel (Microsoft) or Prism (GraphPad).

Sample size was estimated using a DSS research tool to detect effect size of 25% with an α-error of 5% and β-error of 10%. Animals were randomized for tumour experiments into groups such that the average tumour sizes and standard deviations were comparable. Animals that did not have palpable tumours or tumours that were not palpable or measurable were excluded from further analysis at the time of randomization. The tumour measurements were not blinded. Two-tailed Student's *t*-test or Mann–Whitney *U*-test was used to calculate statistical significance. Data that was not normally distributed as assessed by Shapiro-wilk test (Excel, Real statistics add-in) was evaluated using *U*-test. Variance was similar between treatment groups. Survival curves were analysed using a log-rank test. A *P* value<0.05 was considered to be significant.

### Data availability

The authors declare that data supporting the findings of this study are available within the article and the Supplementary Figures.

## Additional information

**How to cite this article:** Wilson, R. *et al*. MicroRNA regulation of endothelial TREX1 reprograms the tumour microenvironment. *Nat. Commun.* **7,** 13597 doi: 10.1038/ncomms13597 (2016).

**Publisher's note**: Springer Nature remains neutral with regard to jurisdictional claims in published maps and institutional affiliations.

## Supplementary Material

Supplementary Information Supplementary Figure 1-16
