# Supplementary material for: MicroRNA regulation of endothelial TREX1 reprograms the tumour microenvironment
Source: Nat Commun. 2016 Nov 25;7:13597. doi: 10.1038/ncomms13597 (PMC5133658; doi:10.1038/ncomms13597)
Supplement: Supplementary Information — Supplementary Figure 1-16 [file ncomms13597-s1.pdf]

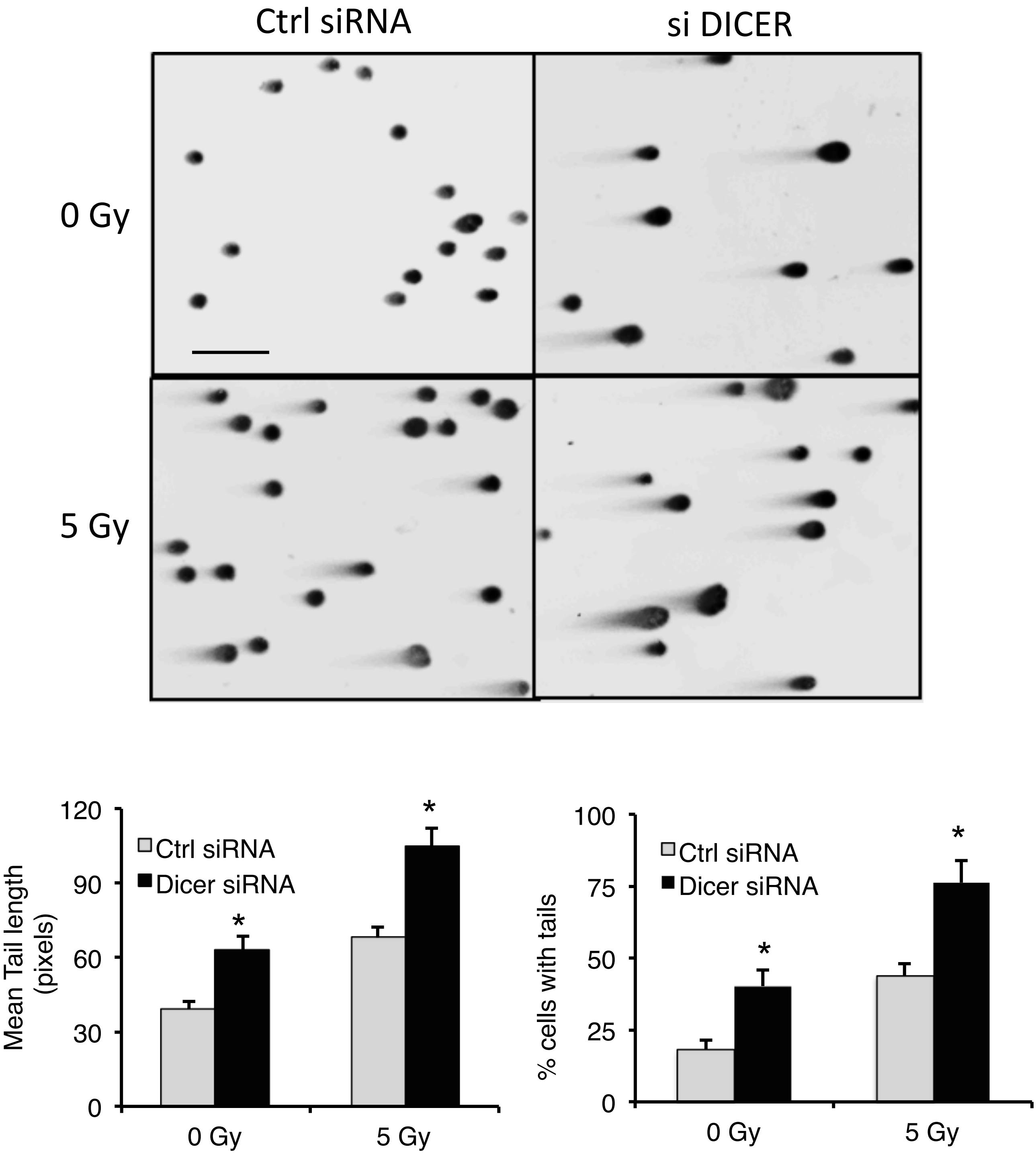

Supplementary Figure 1. Loss of Dicer increases EC DNA damage.
Comet assays of HUVECs with knockdown of DICER and indicated doses of radiation. Bottom panels depict mean+SEM of tail length and % of cells with tails.
* *P*<0.05, two-tailed Student's T-test.

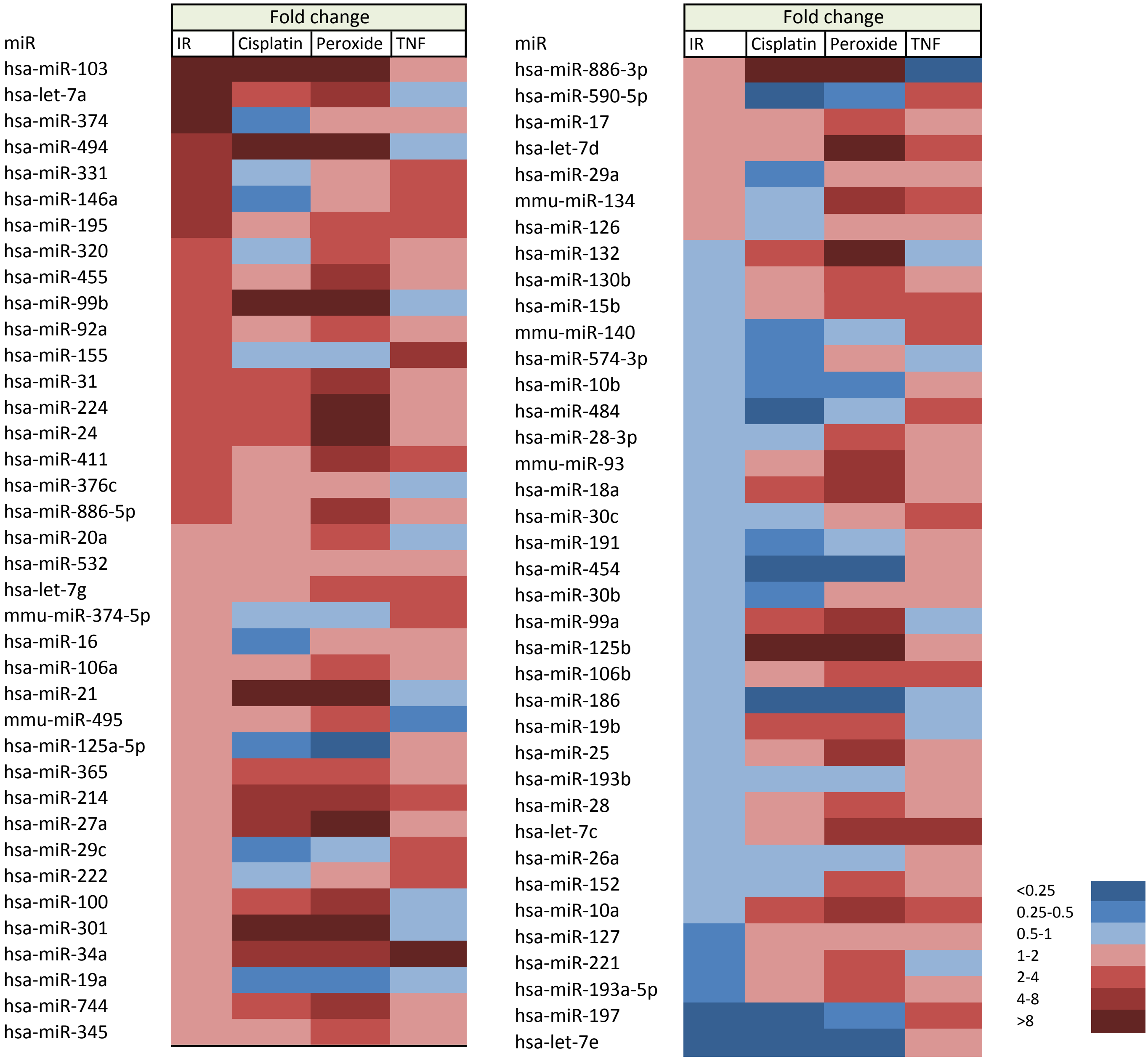

Supplementary Figure 2. Heatmap showing the expression levels of miRs in HUVECs treated with different apoptotic agents.

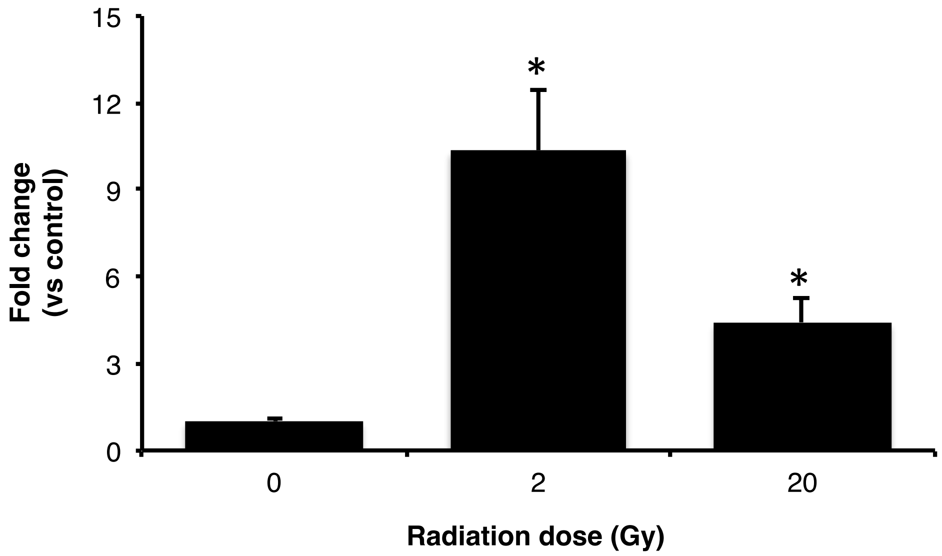

Supplementary Figure 3. Mature miR-103 is induced by both low and high dose radiation.

qRT-PCR of mature miR-103 from HUVECs 1h after the indicated doses of radiation. Mean+SEM of fold change over control is depicted. One of three independent experiments is shown. * P<0.05, student's T-test.

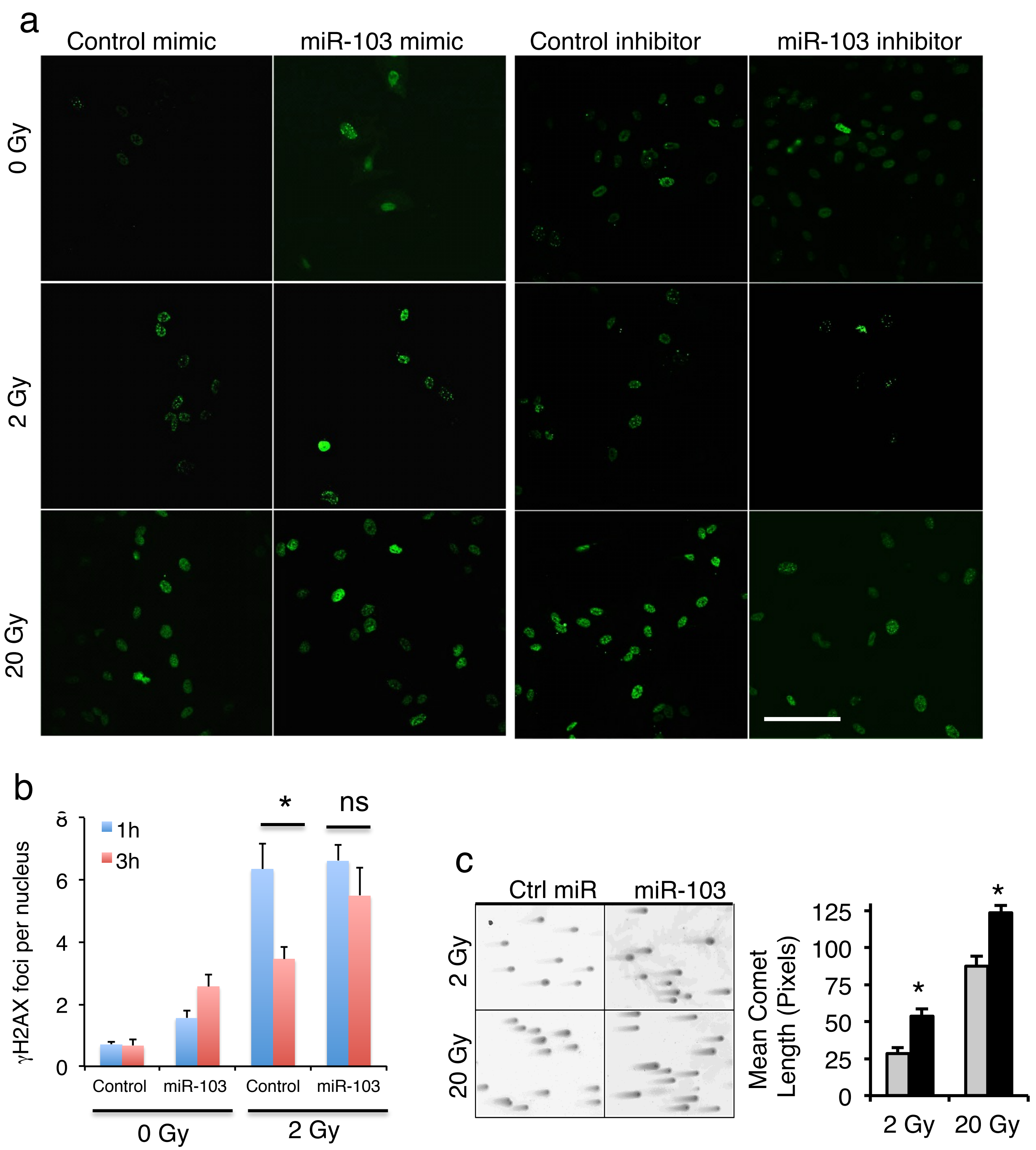

Supplementary Figure 4. miR-103 induces DNA ds breaks in HUVECs
a) Representative immunofluorescence images corresponding to Fig 1g and 1h showing γH2AX foci in HUVECs transfected with the indicated small RNAs treated with either 2 or 20 Gy radiation. These images represent DNA damage 3h post radiation. Scale bar = 100 μm. b) Quantitation of foci for mimics c) A neutral comet assay depicting DNA ds breaks treated with the indicated doses of radiation 24h after ectopic expression of miR-103. Right panel bars show quantification of mean comet lengths from at least 5 different fields. All bars show mean + SEM. * $P<0.05$, Student's T-test

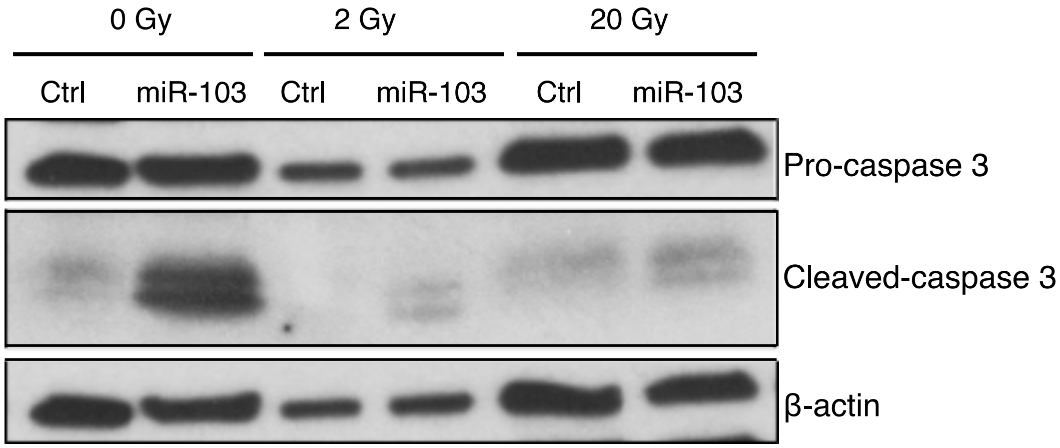

Supplementary Figure 5. Expression of miR-103 activates caspase-3.
HUVECs were transfected with either control mimic or miR-103 mimic for 24h and irradiated at the indicated doses. Caspase-3 activation was analyzed by western blot 24h after irradiation.

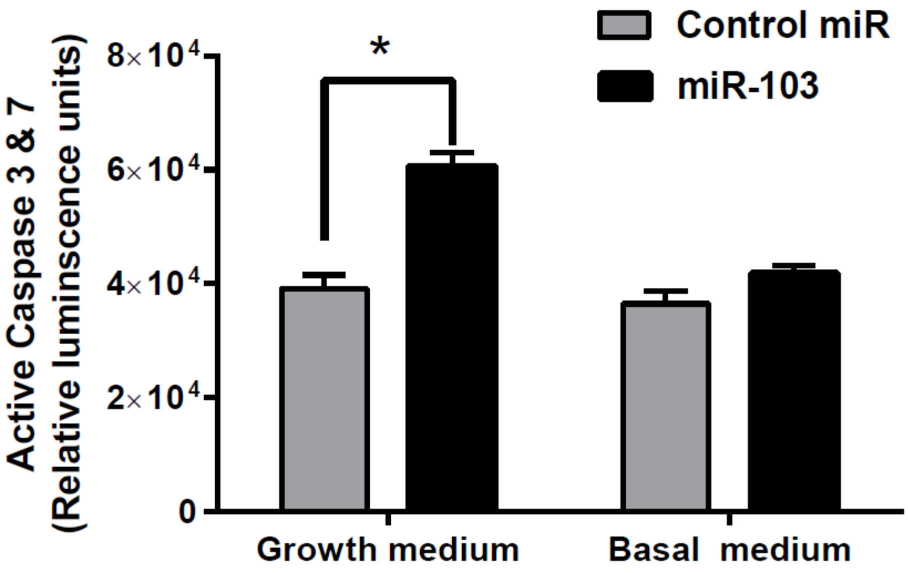

Supplementary Figure 6. miR-103 affects proliferating but not quiescent ECs
HUVECs were transfected with either control mimic or miR-103 mimic and maintained in either regular growth medium with 10% FBS and all growth factors (EGM2 with bullet kit, Lonza) or maintained in basal medium with 1% FBS and no growth factors. Active caspase levels were analyzed by a Caspase-Glo luminescence assay (Promega). Bars show mean + SEM. * $P<0.05$, Student's T-test.

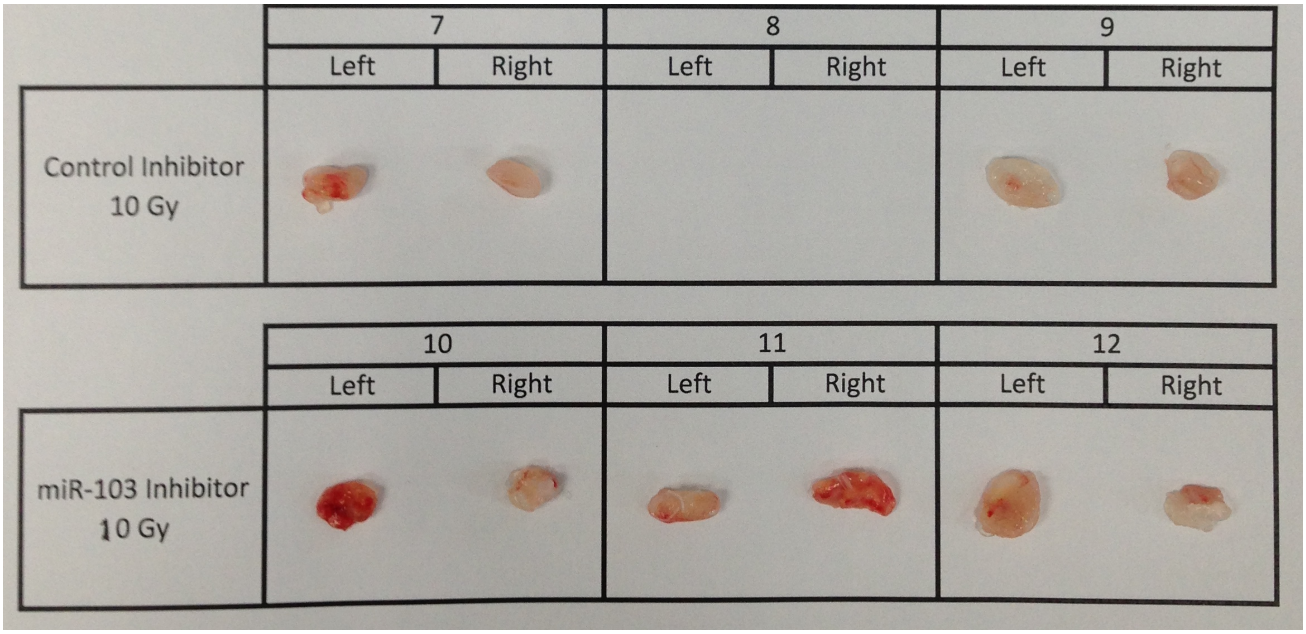

Supplementary Figure 7. Representative images of Matrigel plugs from Fig 2e.
One of two representative experiments is shown.

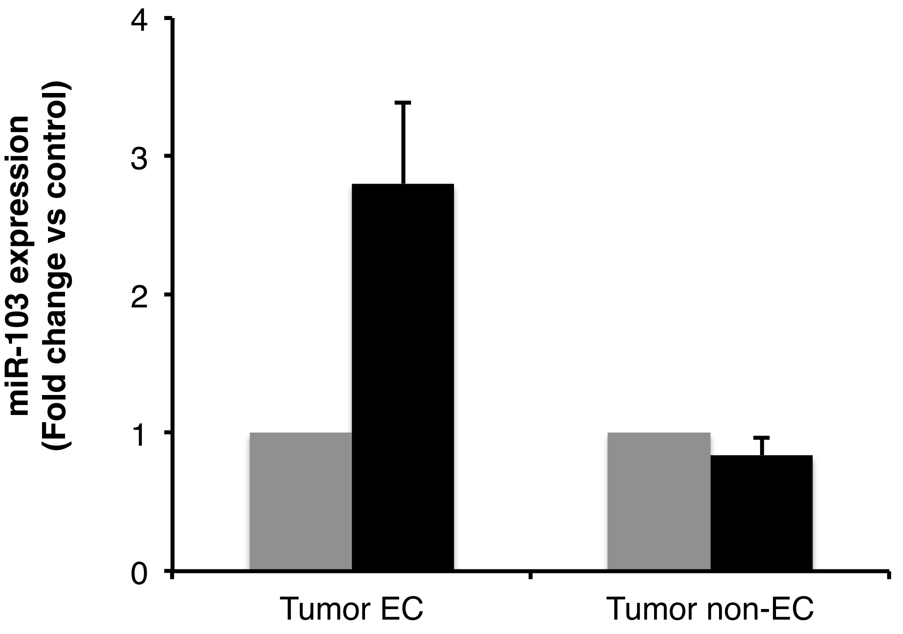

Supplementary Figure 8. 7C1 nanoparticles deliver miR cargo to tumor ECs.

miR expression using qRT-PCR of RNA isolated from the endothelial vs non endothelial cell fractions of an HCT116 tumor suspension. Bars show mean + SEM.

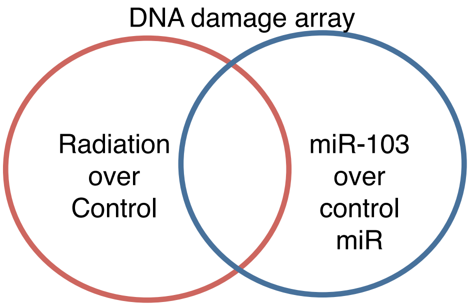

	20 Gy	miR-103
APEX	0.8	1.3
ATM	1.1	1.1
ATR	0.8	1.0
BARD	0.5	1.2
BRCA	0.3	1.1
BRCA2	1.2	1.6
CCNO	0.6	1.1
CHEK1	0.8	1.3
CHEK2	0.6	1.4
DCLRE1A	0.5	1.2
ERCC	0.9	1.2
ERCC2	0.6	1.2
ERCC3	0.8	1.2
ERCC4	1.0	1.1
ERCC5	1.1	1.2
ERCC6	0.7	1.0
ERCC8	0.9	1.4
FANCA	0.4	1.3
FANCC	0.5	1.3
FANCD2	0.8	1.2
FANCE	0.6	1.4
FANCF	0.5	0.6
FANCG	0.7	1.5
FEN1	0.4	1.3
GADD45A	2.1	1.3
GADD45B	0.8	1.1
GADD45G	0.9	1.3
GTF2H1	1.0	1.2
GTF2H3	0.9	1.2
GUSB	1.0	1.3
HPRT1	0.5	1.4
HUS	0.9	1.5
IGF	1.2	1.7
LIG	0.8	1.1
LIG3	0.6	1.3
LIG4	0.9	1.3
MAPK	0.7	1.8
MAPK	0.8	1.3
MAPK	1.8	1.3
MAPK12	0.6	1.2
MAPK8	0.9	1.2
MAPK9	1.0	1.1
MBD4	0.9	1.3
MDM2	5.0	1.1
MGMT	1.1	1.2
MRE11A	0.7	1.3

	20 Gy	miR-103
MSH2	0.6	1.3
MSH3	1.1	1.1
MSH6	0.4	1.2
NBN	0.6	1.2
NTHL1	0.7	1.0
OGG	0.8	1.3
PARP1	0.5	1.0
PCNA	1.0	1.4
PNKP	0.4	1.1
POLA	0.5	1
POLB	0.8	1.2
POLD	0.3	1.1
POLG	0.4	1.2
POLH	1.7	1.1
POLK	1.1	1.3
POLQ	0.8	1.2
POLR	0.6	1.3
POLR	0.8	1.2
POLR2A	0.8	1.4
POLR2B	0.8	1.2
POLR2C	0.6	1.1
PRKDC	0.8	1.2
PSMA3	0.7	1.2
PSMB	0.7	1.3
PSMB5	1.1	1.5
PSMB8	0.9	1.4
PSMB9	0.8	1.1
PSMC4	0.8	1.4
RAD1	0.7	1.4
RAD17	0.9	1.4
RAD23B	0.9	1.3
RAD50	1.1	1.1
RAD51	0.7	1.3
RAD52	0.7	1.3
RAD9A	0.4	1.2
RPA2	0.5	1.1
SMUG1	0.6	1.1
TP53	0.9	1.2
TREX1	0.3	0.5
TREX2	0.3	0.2
XAB2	0.8	1.4
XPA	0.9	1.7
XPC	2.2	1.2
XRCC1	0.8	1.2
XRCC4	1.1	1.7
XRCC5	0.9	1.0
XRCC6	0.6	1.4

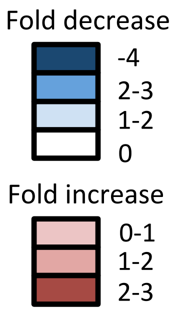

Supplementary Figure 9. Unbiased screen for miR-103 targets in DNA damage pathways.
Heat maps of a DNA damage qPCR array showing normalized mRNA expression levels in HUVECs 24h after treatment with radiation or transfection with miR-103. The fold change levels are calculated over control or mock treated cells.

a

```
                         atgggct cgcaggccct gcccccgggg cccatgcaga ccctcatctt
 841 tttcgacatg gaggccactg gcttgccctt ctcccagccc aaggtcacgg agctgtgcct
 901 gctggctgtc cacagatgtg ccctggagag ccccccacc tctcaggggc cacctcccac
 961 agttcctcca ccaccgcgtg tggtagacaa gctctccctg tgtgtggctc cggggaaggc
1021 ctgcagccct gcagccagcg agatcacagg tctgagcaca gctgtgctgg cagcgcatgg
1081 gcgtcaatgt tttgatgaca acctggccaa cctgctccta gccttcctgc ggcgccagcc
1141 acagccctgg tgcctggtgg cacacaatgg tgaccgctac gacttccccc tgctccaagc
1201 agagctggct atgctgggcc tcaccagtgc tctggatggt gccttctgtg tggatagcat
1261 cactgcgctg aaggccctgg agcgagcaag cagcccctca gaacacggcc caaggaagag
1321 ctacagccta ggcagcatct acactcgcct gtatgggcag tcccctccag actcgcacac
1381 ggctgagggt gatgtcctgg ccctgctcag catctgtcag tggagaccac aggccctgct
1441 gcggtgggtg gatgctcacg ccaggccttt cggcaccatc aggcccatgt atggggtcac
1501 agcctctgct aggaccaagc caagaccatc tgctgtcaca accactgcac acctggccac
1561 aaccaggaac actagtccca gccttggaga gagcaggggt accaaggatc ttcctccagt
1621 gaaggaccct ggagccctat ccagggaggg gctgctggcc ccactgggtc tgctggccat
1681 cctgaccttg gcagtagcca cactgtatgg actatccctg gccacacctg gggagtaggc
1741 caagaaggaa aatctgacga ataaagaccc ccgctgcccc ataaaaaaaa aaaaaaaaaa
1801 aaaaaaaaaa aaaaaaaaaa a
```

b

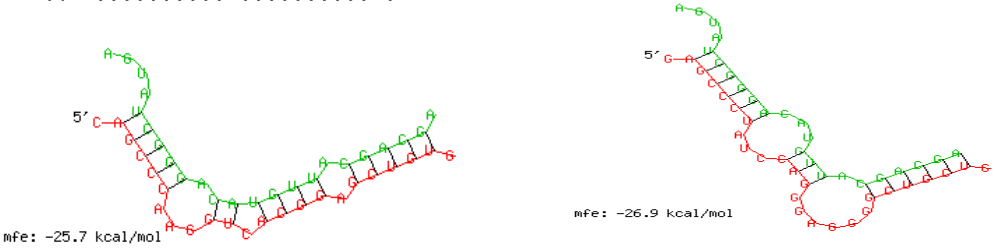

c

```
1141 cctcagttct gtataggcaa tgctgtgtta ttacttgaat atagaatata tagtttacaa
1201 aatgaaaatt acaatgttct caccaaatat atgccttcgt gtgtccaaag tataattatt
1261 ttagatgcta attttgaata gtttattaaa cagttataaa tatgcaaagt agctggcatg
1321 tagtgtcacg gattttctgg atagaggaag tgattggaag tactccactt aaagccatgg
1381 aattagcaat agtttgcttt ttaatagaag gcccatttgt aagaatgttg aaaatatgtg
1441 taccgtttaa agaaaaagca gctttaaagt gacaaacaaa ataccctttt tcttttagta
1501 tggtttattt ttctaggttt tctgtccctc cctcagtagt gaagagtttt ctttattcct
1561 ggcagtgtca ggaatattgg tttgaaaagc tgttggccta tctggagttt ggccttgtta
```

d

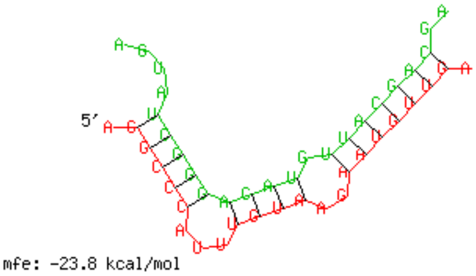

Supplementary Figure 10. RNA hybrid models of miR-103 binding sites
(A) mRNA sequence of human TREX1 showing miR-103 binding sites in the coding region. (B) RNA hybrid modeling of the miR-103 TREX1 interaction reveals high binding energies for the two sites. (C) mRNA sequence of human FANCF showing the stop codon and miR-103 binding site. (D) RNA hybrid modeling of the miR-103-FANCF interaction. miR binding sites are conserved between human and mouse TREX1 and FANCF.

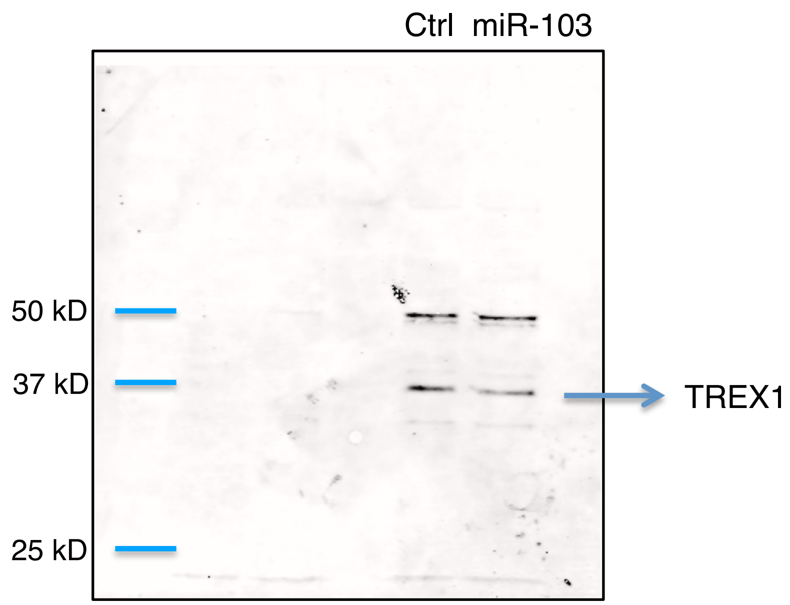

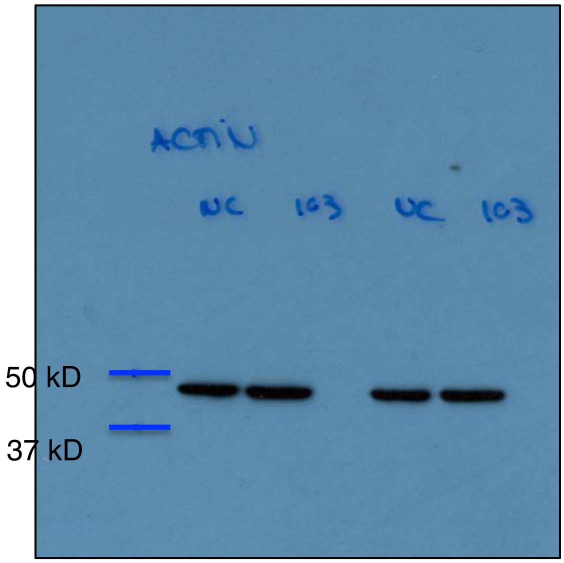

Supplementary Figure 11. Full lane images of western blots from figure 3

TREX 1 A	ctggagccctatccagggaggggctgctggcc
TREX 1 A Mut	ctggagaaatat ccagggaggg aaataaggcc

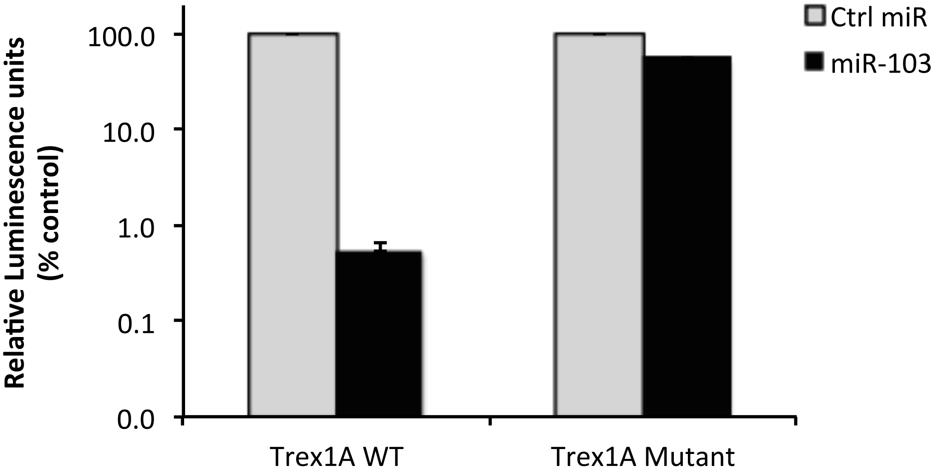

Supplementary Figure 12. TREX1 A site harbors a specific binding region for miR-103. Top panel shows the binding region of miR-103 on TREX1 coding region (green shaded) and the mutations (grey shaded). Luminescence from 3'UTR-luciferase constructs with either WT or mutant miR-103 binding regions 24h after transfection with Control miR or miR-103. Bars show mean + SEM.

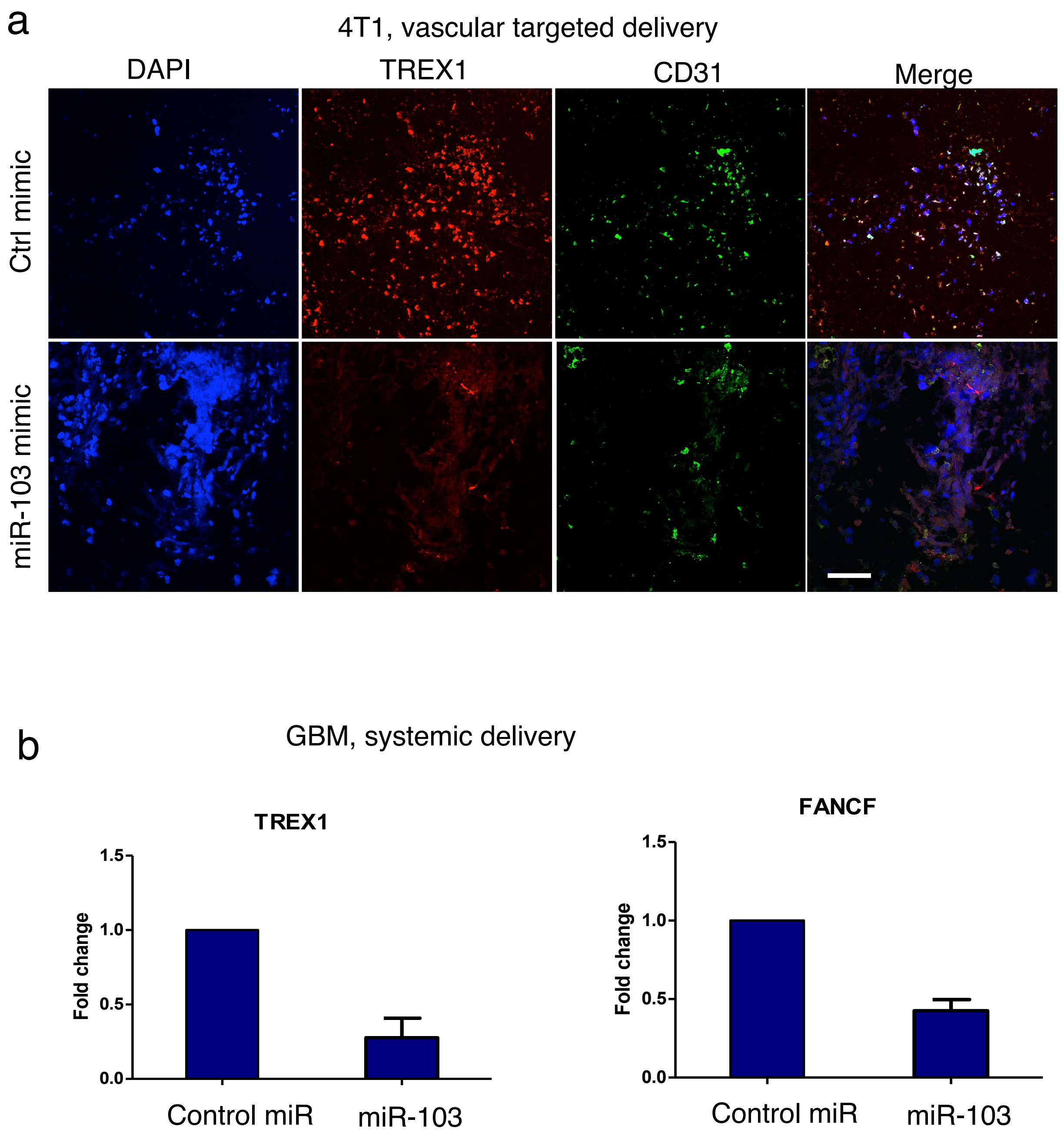

Supplementary Figure 13. miR-103 treatment decreases endothelial TREX1 in vivo. a) Representative Immunofluorescence images from sections stained for TREX1 and CD31 from 4T1 tumors treated with either miR mimic or miR-103 in 7C1 nanoparticles (Fig 3 f-g). Scale bar = 100 µm. b) Nu/Nu mice were injected with GBM-NS-001 cells ($1x10^6$ cells) subcutaneously. 20 days post injection, the mice were randomized to miR control or miR103 (n=5 per group and 15 nanomoles of miR was injected i.v. Tumors were harvested 48h later and endothelial cells were sorted as CD45-CD31+CD34+ followed by RT-PCR analysis. Bars show mean + SEM.

	24h		48h		72h	
	miR-103	miR-103+TREX1 Protector	miR-103	miR-103+TREX1 Protector	miR-103	miR-103+TREX1 Protector
IP-10	38.9	1.5	60.6	1.0	53.9	0.6
RANTES	13.5	3.9	74.5	2.3	63.9	0.8
IL-15	8.3	1.0	8.3	2.5	4.5	1.0
MIG	7.0	7.0	1.0	0.1	7.0	1.0
MIP-1alpha	3.4	2.2	2.2	2.2	1.6	1.0
IL-12	2.7	0.0	1.0	0.0	7.4	0.0
INF-gamma	2.4	2.4	2.4	0.5	3.3	0.7
IL-13	1.9	0.0	49.8	49.8	0.0	0.0
MCP-1	1.5	0.2	1.6	0.1	1.0	0.0
IL-2	1.4	1.2	1.2	1.0	1.4	1.0
IL-6	1.3	2.2	0.8	0.6	0.5	0.1
MIP-1beta	1.3	1.0	1.0	1.0	1.3	1.0
IL-4	1.2	1.0	0.8	1.0	1.0	0.7
IL-1RA	1.2	0.9	1.0	1.1	1.1	1.0
IL-17	1.2	1.2	0.9	0.9	1.2	1.0
IL-10	1.0	0.9	1.0	1.0	1.0	1.0
GM-CSF	1.0	0.9	0.9	0.9	0.7	0.7
INF-alpha	1.0	1.0	1.0	1.0	1.0	0.1
TNF-alpha	1.0	2.0	3.3	1.0	2.0	1.0
IL-7	1.0	1.0	3.4	0.8	33.1	1.0
IL-2R	1.0	1.5	1.5	1.0	1.0	1.0
IL-5	0.9	0.9	1.0	1.0	0.9	1.0
Eotaxin	0.9	1.5	1.2	0.8	1.0	1.0
IL-1Beta	0.5	0.5	0.5	1.0	1.0	1.0
IL-8	0.4	0.2	0.4	0.1	0.1	0.0

Supplementary Figure 14. Secretome induced by miR-103 expression.
HUVECs were transfected with miR-103 or a control miR and supernatant was harvested at the indicated time points. The supernatants were analyzed for cytokine expression using a human cytokine/chemokine multiplex luminex assay. Heatmap depicts fold change in miR-103 treated supernatants with or without TREX1 target protectors vs control miR supernatants.

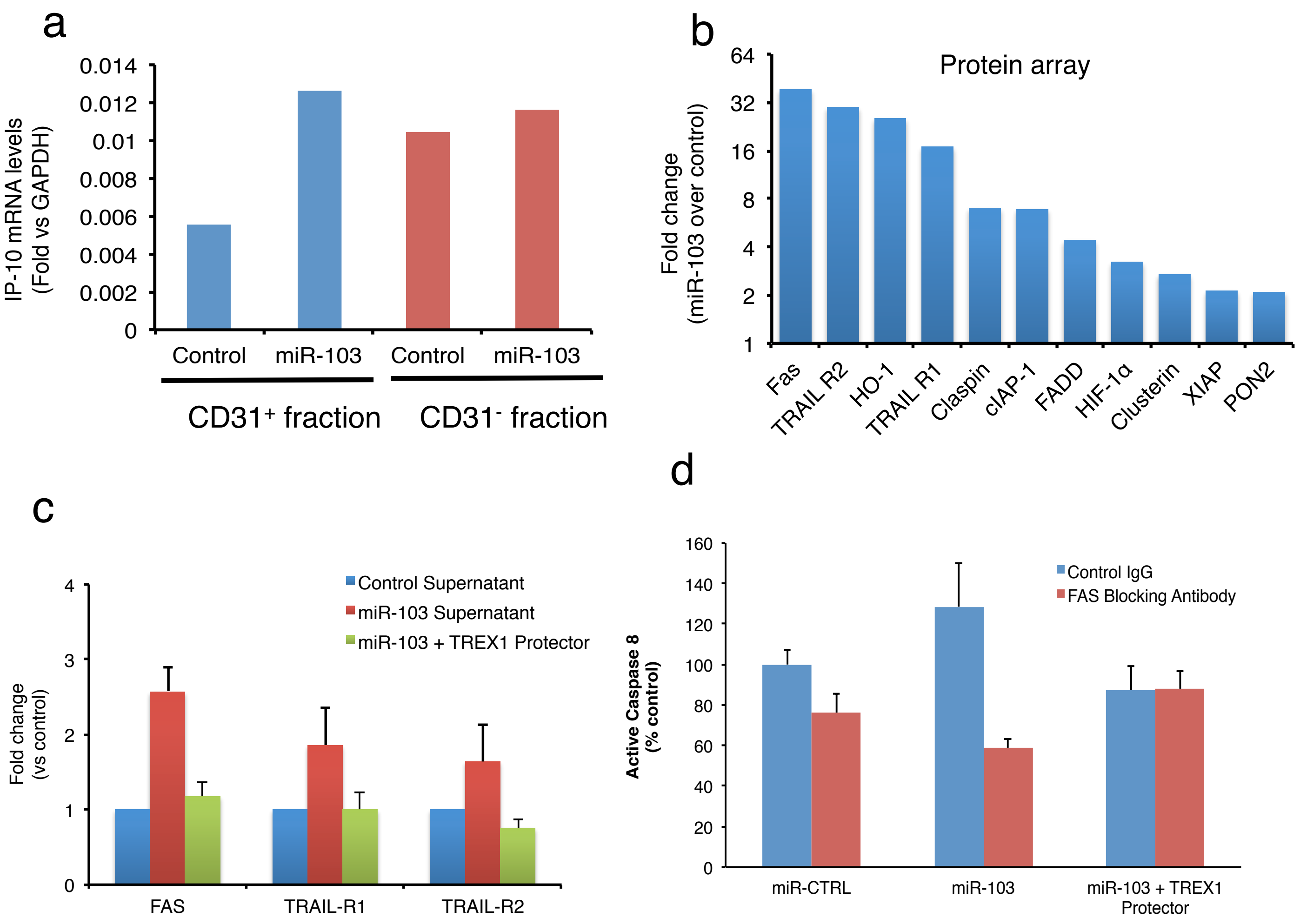

Supplementary Figure 15. miR-103 causes tumor cell death in a paracrine fashion. (a) Upregulation of IP10 mRNA in tumor endothelial cells after 7C1 delivery of miR-103 in HCT116 xenografts. Day 17 tumors from Fig 3c were used for this assay. (b) Protein expression on a cell survival membrane array using whole tumor lysate of a HCT116 tumor 48h after a single miR-103 injection compared to control miR injection. (c) Gene expression in HCT116 cells after 72h of culture with conditioned media from HUVECs transfected with either Control miR, miR-103 or miR-103+TREX1 target protector. Bars depict mean + SD fold change. (d) Active Caspase-8 levels of HCT116 cells 72h after treatment with conditioned media from HUVECs transfected with the indicated oligos. Bars depict mean + SD % change over control miR transfected, control IgG treated cells.

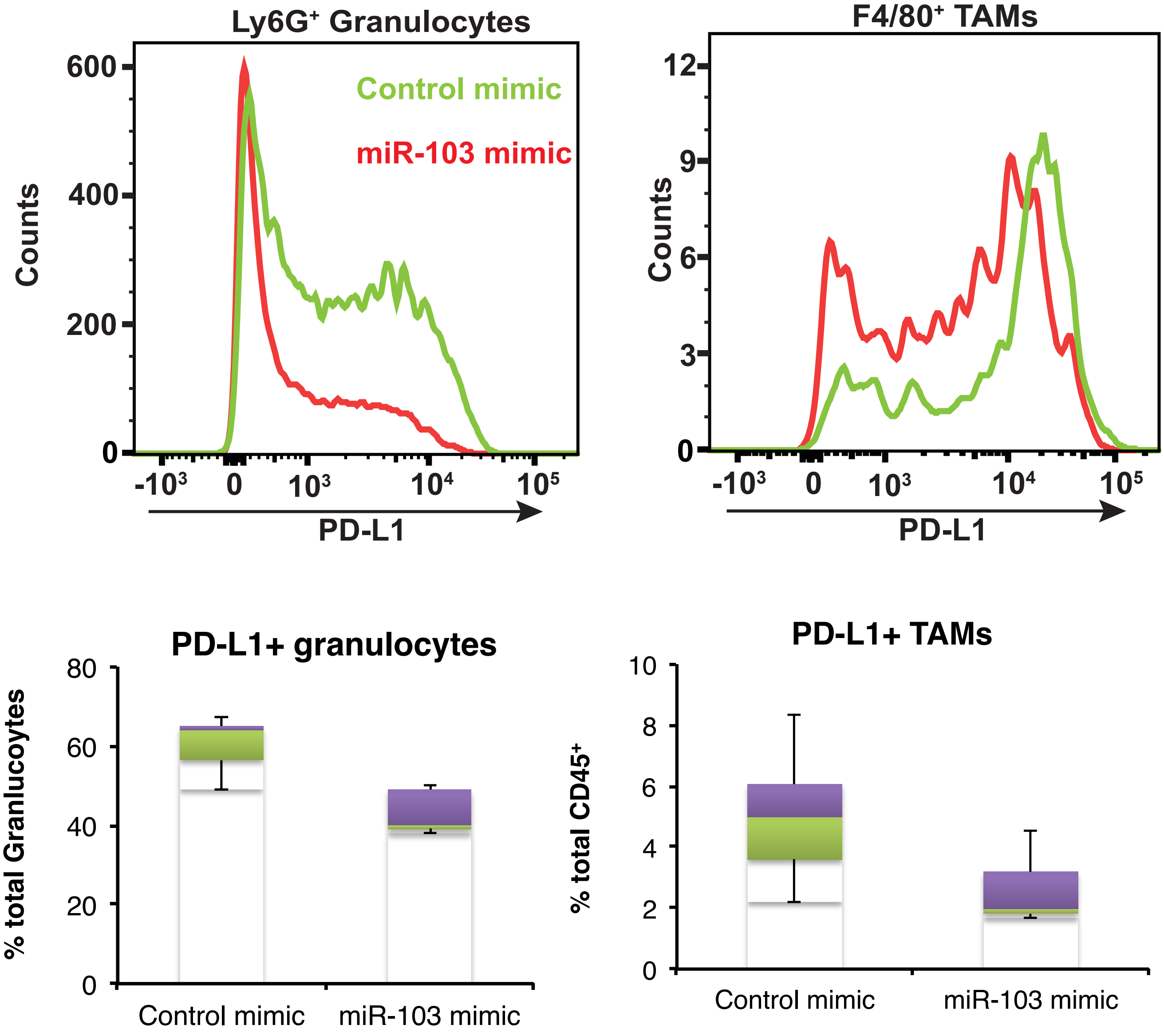

Supplementary Figure 16. miR-103 treatment affects the immune microenvironment Representative flow cytometry histogram plots and box plots of PD-L1 expression on Ly6G+ granulocyte population and F4/80+ tumor associated macrophages from 4T1 tumor bearing mice treated with either control mimic or miR-103 mimic as described in Fig 3f. (n=3 mice per group). Bars show interquartile range.
